# Characterization of alginate-degrading bacteria isolated from seaweed-producing areas of South Korean territory and verification of the bacteria as plant growth-promoting biofertilizer

**DOI:** 10.1128/spectrum.03164-24

**Published:** 2025-04-29

**Authors:** Seung Hwa Jeong, Pyeong Ho Lee, Joon Kwan Moon, Sujin Lee, Yeonjong Koo

**Affiliations:** 1 Department of Agricultural Chemistry, Chonnam National University 34931, Gwangju, Republic of Korea; 2 Horticultural and Herbal Crop Environment Division, National Institute of Horticultural and Herbal Science, Rural Development Administration, Wanju, Republic of Korea; 3 School of Plant Resources and Landscape Architecture, Hankyong National University 105909, Anseong, Republic of Korea; 4 Department of Environment and Energy Engineering, Gwangju Institute of Science and Technology 65419, Gwangju, Republic of Korea; 5 Department of Biomaterial Convergence, Chonnam National University 34931, Gwangju, Republic of Korea; Instituto de Ecología, A.C. (INECOL), Pátzcuaro, Michoacán, Mexico

**Keywords:** alginate, seaweed utilization, alginate-degrading bacteria, alginate lyase, PGPR

## Abstract

Alginate, a carbohydrate polymer produced by seaweed and some bacteria, is widely used in the food and medicine industries. Alginate oligosaccharides, produced by depolymerizing alginate, exhibit diverse biological functions, such as plant protection and growth promotion, enhancement of microbial metabolic activity, and potential applications in biofuel production. This study isolated 13 alginate-degrading bacterial strains from over a thousand seaside soil bacteria in South Korea and investigated their alginate-degrading characteristics. Through 16S rDNA sequencing, *Marinomonas sp*., *Zobellella sp*., and *Pseudomonas sp*. were newly identified at the subspecies level. Notably, this study is the first to report the alginate-degrading capability of *Zobellella sp*. The substrate specificity of each bacterial strain was analyzed toward poly-α-L-guluronate and poly-β-D-mannuronate, the two major alginate polymeric components. Furthermore, 3 of the 13 isolated strains were demonstrated to synthesize auxin, and their application to *Arabidopsis thaliana* confirmed enhanced vegetative growth. These findings advance our understanding of alginate degradation and highlight the potential of these bacteria and their alginate lyases as valuable resources in biotechnology applications. In addition, the potential of these bacteria as biofertilizers for promoting plant growth and the production of functional alginate oligosaccharides underscores the need for further exploration and development in this field.

**IMPORTANCE:**

This study aimed to isolate alginate-degrading bacteria from the soil samples collected in South Korea’s major seaweed production areas and evaluate their potential as biofertilizers. Alginate, a primary component of brown algae, breaks down into alginate oligosaccharides, which are known to enhance plant growth. In this study, 13 strains of alginate-degrading bacteria were isolated, and some of them showed the potential for plant growth promotion and stress defense through strong biofilm formation and auxin production. Importantly, these bacterial strains exhibited plant growth-promoting potential, demonstrating their applicability in combination with seaweed-based fertilizers. These findings provide valuable insights that could broaden the industrial utilization of seaweed-derived fertilizers, contributing to enhanced agricultural productivity and sustainability.

## INTRODUCTION

Alginate lyase is an enzyme that breaks down the polysaccharide alginate, a significant component of the cell wall of brown seaweed (1–3). Alginate lyase-producing bacteria have been isolated from marine environments, including sediments, seawater, algae, and microorganisms (1, 4–6). *Pseudomonas aeruginosa*, a gram-negative bacterium commonly found in soil, water, and plants (7, 8), *Vibrio* sp. strain QY105 isolated from a sea mud sample (9), and *Cellulophaga algicola* isolated from marine diatoms showed alginate lyase activity (10, 11). *Flavobacterium* sp. strain UMI-01 isolated from a seaweed sample exhibited alginate lyase and laminarinase activities (12–15).

Given that brown seaweed is an abundant and sustainable source of biomass, extracellular alginate lyase has several potential applications in various industrial fields, including the production of biofuels (16–18). Alginate lyase hydrolyzes alginate to monomeric sugars, which can be fermented to produce biofuels such as ethanol or butanol. It is a sustainable and cost-effective alternative to traditional biofuel production. Alginate lyase is also widely used in the pharmaceutical industry. For example, alginate is used to carry drugs or proteins in drug delivery systems due to its biocompatibility, biodegradability, and non-toxicity. Enzymatic alginate degradation by alginate lyase allows targeted delivery and controlled release of drugs (3, 19, 20). Alginate lyase can be applied in bioremediation to break down seaweed debris (21, 22). This enzymatic activity mitigates the environmental impact of marine biofilms and debris and maintains a more sustainable aquatic ecosystem.

Alginate lyase-producing bacteria enhanced the growth and yield of various crops, including tomato, maize, lettuce, and banana (23–26), highlighting their potential in agriculture. This is mainly due to the ability of these bacteria to break down alginate and release nutrients available to plants (23). Alginate lyase-producing bacteria also contribute to plant defense by producing bioactive compounds that protect plants from pathogens. For example, *Pseudomonas aeruginosa* produces phenazines with antifungal and antibiotic properties (27). Alginate lyase-producing bacteria also help plants tolerate environmental stresses, such as drought or salinity (28–31).

Genes encoding alginate lyase were identified in various bacteria, including *Pseudomonas aeruginosa* (8, 32), *Vibrio* sp. (9, 33, 34), *Cellulophaga* sp. (35, 36), and *Flavobacterium* sp. (37, 38). They are found in plasmids or the bacterial chromosome. Alginate lyases vary in size from 23 to 100 kDa and are typically composed of a signal peptide, a catalytic domain, and a carbohydrate-binding module (1). The catalytic domain of alginate lyase contains conserved amino acid residues that are critical for cleaving the glycosidic bond between the mannuronate or guluronate residues in alginate (39, 40). The carbohydrate-binding module is responsible for binding to the alginate substrate and positioning the enzyme for cleavage.

In the present study, we successfully isolated 13 alginate-degrading bacterial strains with varying enzymatic activities. Notably, three of these strains were newly identified for their ability to degrade alginate. The isolates were also evaluated for their plant growth-promoting capacity and applicability in agricultural fields. Overall, this report presents the identification of multiple alginate lyase-producing bacteria along with a comprehensive characterization of their enzymatic properties, providing valuable insights into their potential biotechnological applications.

## MATERIALS AND METHODS

### Soil collection and bacterial isolation

Seaside soils were collected from Goha Island in Yudal-dong, Mokpo-si (34°46′26.2″N, 126°21′27.7″E), 1736 Dodu-dong, Jeju-si (33°30′32.7″N, 126°28′12.7″E), 2812-9 Hagwi 2-ri, Aewol-eup, Jeju-si (33°29′21.1″N, 126°23′25.5″E), Aewol Coastal-ro, Aewol-eup, Jeju-si (33°28′05.6″N, 126°18′59.2″E) and 62-22 Sirang-ri, Gijang-eup, Gijang-gun, Busan (35°11′50.7″N, 129°13′45.6″E). 0.1% (wt/vol) of sodium alginate (sodium alginate from brown algae, Sigma) was added to promote the growth of alginate-degrading microorganisms in each soil sample. The samples were mixed by vortexing and sedimented gravimetrically for 24 hours. The interspace between the soil and water layers was taken for isolating bacteria. Trypticase soy agar-yeast extract (TSA-YE), alginate minimal (A+; composed of 0.4% sodium alginate (0.016 mM), 0.17 M sodium chloride, 1 mM peptone, and 5 mM bacto agar), and Luria-Bertani (LB) media were used as the bacterial culture media.

### Screening for alginate-degrading bacteria

To select bacteria that degrade alginate, the reducing sugar quantification method using 3,5-dinitrosalicylic acid (DNS) was modified and utilized (41). Colonies formed on the TSA-YE, A+, and LB agar media were inoculated into 0.4% (wt/vol) alginate minimal medium and cultured at 30°C for 18 hours in a 96-well microplate. After centrifugation at 6,000 rpm for 1 minute, the supernatant and the DNS reagent were mixed at a ratio of 1:1 (vol/vol) and then reacted at 95°C for 10 minutes. Absorbance at 540 nm was measured using a Microplate Reader (BioTek, US/Epoch) to quantify the colorimetric change from yellow to orange or red, and reducing sugars produced were calculated based on a standard curve for glucose (Fig. S1).

### Identification of bacteria

Genomic DNA was extracted from the isolated bacteria using the Accuprep Genomic DNA Extraction Kit (Bioneer). The 16S rDNA gene was amplified using universal primers 27F (5′-AGAGGTTTTGATCMTGCTCAG) and 1492R (5′-TACGYTACCTTGTACGAGACTT) and analyzed by Sanger sequencing. Sequence results were used to identify microorganisms in the 16S rDNA database of the National Center for Biotechnology Information (NCBI) BLAST. The 16S rDNA sequence of the isolated strains and the sequence of the genetically close reference strain were aligned using the ClustalW system of MEGA11. A phylogenetic tree was generated using a neighbor-joining algorithm and validated with 1,000 bootstrap replicates.

### Quantification of alginate-degrading activity

The DNS assay, which measures the amount of reducing sugars, was employed to quantify the alginate degradation activity of bacteria. Lysing activity by a bacterium was defined as the amount of reducing sugars produced normalized to bacterial growth.

### Verification of alginate lyase secretion

The alginate-degrading bacteria were inoculated into 0.1% (wt/vol) alginate minimal liquid medium to reduce the influence of alginate in microbial culture and cultured at 30°C for 18 hours. After the OD (A_600_) was measured, 1 mL of the culture was centrifuged at 6,000 rpm for 1 min. The pellet and supernatant were separated to examine alginate lyase’s intracellular and extracellular activities, respectively. The pellet was sonicated in the PBS solution to prepare the cytosolic protein extracts, and the cell debris was removed by centrifugation at 12,000 rpm for 10 min. The cytosolic solution or the supernatant was reacted with 0.2% (wt/vol) of alginate solution in the reaction buffer (10 mM Tris-HCl [pH 8.0], 0.1 M NaCl) at 37°C. After 24 and 48 hours of reaction, the reducing sugar contents were measured by DNS assay as described previously. The experiments were repeated three times.

The Bradford assay was performed to quantify the proteins in the supernatant using the Pierce Bradford Protein Assay Kit (Thermo Fisher) (42).

### Substrate specificity test

To determine the substrate specificity of the alginate-degrading bacteria, sodium alginate, β(1→4) D-mannuronate oligosaccharides (poly-M, Mannuronic Block OLS. DP20-DP35, Elicityl) and α(1→4) Guluronate oligosaccharides (poly-G, Guluronate Block OLS. DP25-DP45, Elicityl) were tested. Bacteria were grown in the 0.1% (wt/vol) alginate minimal medium at 30°C for 18 hours. The bacterial growth was measured by absorbance at 600 nm, and the supernatant was obtained by centrifugation at 6,000 rpm for 1 minute. The supernatant was reacted with the final 0.2% (wt/vol) of each substrate in the reaction buffer (10 mM Tris-HCl [pH 8.0], 0.1 M NaCl) at 37°C for 4 hours (43). The reducing sugar contents were quantified by DNS assay.

### Biofilm synthesis test

Bacterial biofilm formation by the alginate-degrading bacteria was colorimetrically detected using the Quanti-Micro Biofilm Biomass Formation Assay Kit (Biomax) (44, 45). Briefly, the bacterial culture diluted 100-fold in TSB medium containing 1% glucose was dispensed into each well of a 96-well plate and cultured with a 96-peg lid on at 37°C for 24 hours. The 96-peg lid was carefully washed in 1× PBS twice, stained with crystal violet solution for 15 minutes, and washed again in 1X PBS. After incubation in 95% ethanol at room temperature for 30 minutes, the biofilm formation was quantified by measuring the absorbance at 590 nm using a plate reader. The biofilm formation ability of bacteria was classified into weak, moderate, or strong, according to the manufacturer’s instructions.

### Detection of auxin production

Auxin production by the alginate-degrading bacteria was detected by the colorimetric analysis using the Salkowski reagent (46). First, the bacteria were cultured in the LB medium supplemented with 0.5 mM of L-tryptophan at 30°C for 18 hours. The supernatant was reacted with a Salkowski reagent (0.5 M FeCl_3_ in 35% HClO_4_ in a proportion of 1:50 [vol/vol]) for 30 minutes in dark conditions. The absorbance at 535 nm was measured using a plate reader.

To identify Indole-3-acetic acid (IAA), a liquid chromatography-tandem mass spectrometry (LC-MS/MS) assay was performed on an LC/MS-8040 (Shimadzu). The bacterial culture’s supernatant was injected into a packed column (Kinetex C18, 150 × 2.1 mm, 1.7 µm, Phenomenex) and eluted with a 0.3 mL/min flow rate at 40°C (47).

### Assessment of plant growth promotion by alginate-degrading bacteria

*Arabidopsis thaliana* (Col-0) seeds were sown in the horticulture soil (Hungnong Bio Co. Ltd.) and grown at 22°C under long-day conditions (16 h light/8 h dark) for a week. The bacterial strains were grown in 5 mL of LB medium and harvested when A_600_ reached 0.5 by centrifugation at 6,000 rpm at 4°C for 5 minutes. The cell pellet was resuspended in 5 mL of 0.4% (wt/vol) sodium alginate solution and treated over the soil around the plant weekly. After 5 weeks of growth, the phenotype parameters of leaves, such as fresh weight, dry weight, number of leaves, and total leaf area, were measured. Statistical analysis was conducted using one-way ANOVA followed by Tukey’s Honest Significant Difference (HSD) test for multiple comparisons.

## RESULTS

### Isolation and identification of alginate-degrading bacteria

South Jeolla Province, Jeju Island, and South Gyeongsang Province are major seaweed production regions in South Korea. To isolate alginate-degrading bacteria, coastal soil samples were collected from the five sites in these areas. 0.1% (wt/vol) of sodium alginate was added to the soil samples to outgrow alginate-degrading bacteria. A total of 61 single colonies were cultured and screened for their ability to grow in the alginate minimal medium. Thirteen candidates from 61 colonies showed a color change from yellow to red or orange by the DNS assay, indicating these bacteria’s production of reducing sugars (Fig. S1B). The amount of reducing sugars was estimated based on the absorbance at 540 nm (Table 1).

**TABLE 1:** Thirteen species of alginate-degrading bacteria identified^*a*^

Isolation	Target organism for identification	16S rDNA homology (%)	GenBank accession number (16S)	[Red. sugar] (mg/mL)	GenBank accession number (genome)
A2-20	*Stenotrophomonas maltophilia*	98.94	NR112030	0.229	CP173765
B1-1	*Vibrio algivorus*	99.86	NR151933	0.448	CP168145– CP168149
B2-2	*Bacillus toyonensis*	99.86	NR121761	0.029	
B3-2	*Vibrio alginolyticus*	99.65	NR118258	0.100	CP167181– CP167184
C1-2	*Vibrio natriegens*	99.16	NR117890	0.869	CP167179– CP167180
C3-3	*Marinomonas polaris*	98.32	NR042340	0.055	CP165966
D8-2	*Zobellella aerophila*	98.80	NR117862	0.045	CP165965
D8-3	*Bacillus manliponensis*	99.86	NR125530	0.040	
D6-3	*Pseudomonas khazarica*	99.86	NR169334	0.034	
D9-1	*Lysinibacillus pakistanenesis*	99.09	NR113166	0.078	CP165964
E1-3	*Stenotrophomonas cyclobalanopsidis*	99.37	NR180613	0.073	
E1-YE2	*Stenotrophomonas nematodicola*	99.72	NR181111	0.206	CP165963
E1-LB2	*Aeromonas salmonicida*	99.79	NR043324	0.141	

^*a*^ The closest related strains were identified based on 16S rDNA sequences, with their corresponding GenBank accession numbers provided. Sequence identity values are presented as measures of homology. The reducing sugar production capabilities observed during the screening process are also shown.

**Fig 1:**
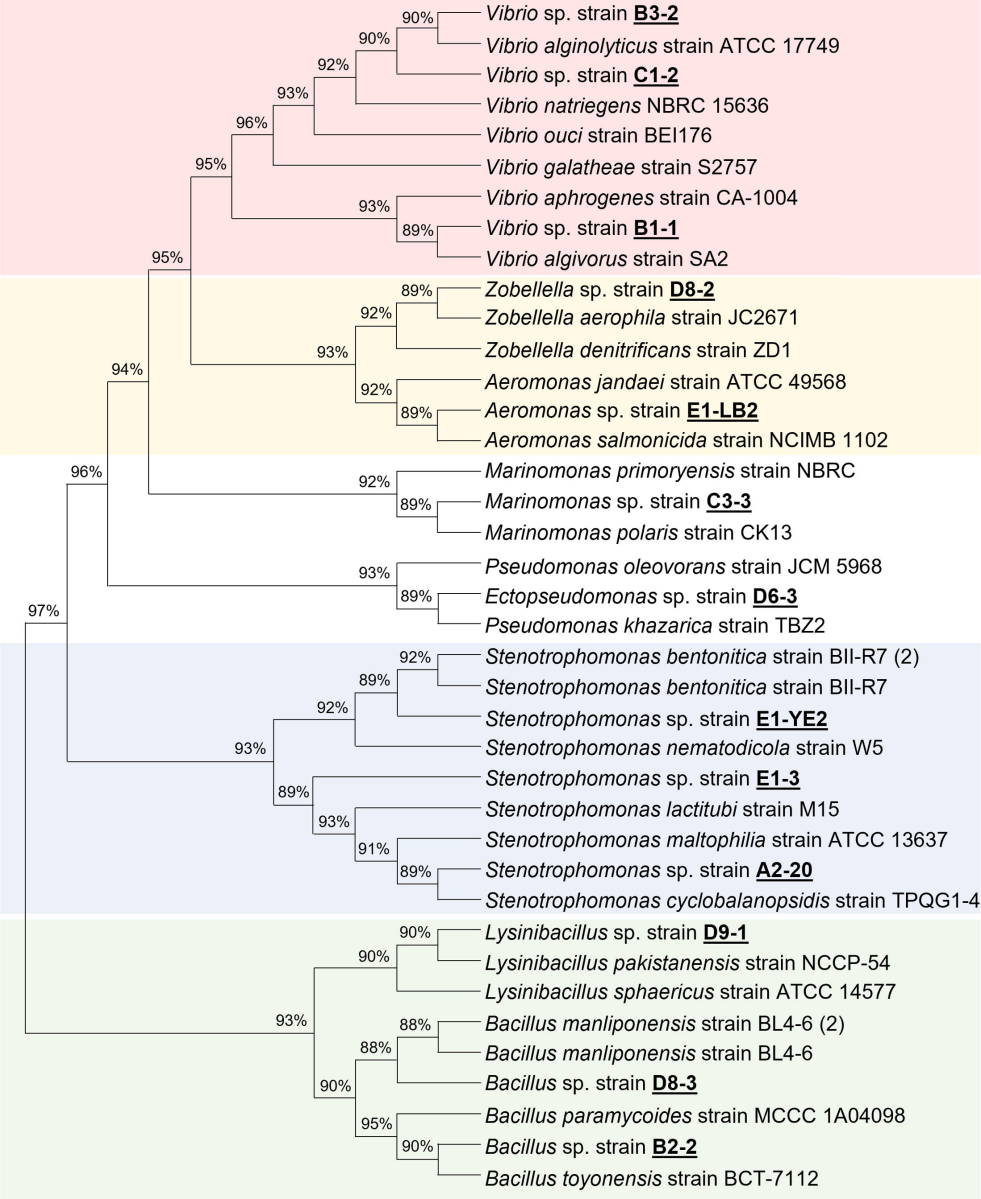
Phylogenetic tree of alginate-degrading bacteria. A phylogenetic tree of the isolated alginate-degrading bacteria was constructed using the neighbor-joining method in MEGA11, based on 16S rDNA sequences. Bootstrap values greater than 50% (from 1,000 replicates) are shown at each node. Different background colors were applied to distinguish each genus.

The 16S rDNA gene was amplified from the genomic DNA and sequenced to identify these candidate isolates (Table S1). 16S rDNA sequencing analysis identified 13 bacterial species belonging to seven genera: *Vibrio*, *Zobellella*, *Aeromonas*, *Marinomonas*, *Pseudomonas*, *Stenotrophomonas,* and *Bacillus* (Table 1 and Fig. 1). *Stenotrophomonas sp*. strain A2-20, *Marinomonas sp*. strain C3-3, and *Zobellella sp*. strain D8-2 showed 16S rDNA sequence homology lower than 99%, suggesting they are likely new bacterial subspecies. Previous studies revealed the presence of alginate degradation activity or alginate lyase for the genera *Stenotrophomonas*, *Vibrio*, *Bacillus*, *Marinomonas*, *Pseudomonas*, and *Aeromonas* (9, 43, 48–51). Remarkably, the alginate-degrading activity of *Zobellella* sp. strain D8-2 was demonstrated for the first time in this study, highlighting the need for further research to isolate valuable microbial resources.

### Evaluation of bacterial alginate-degrading activity

**Fig 2:**
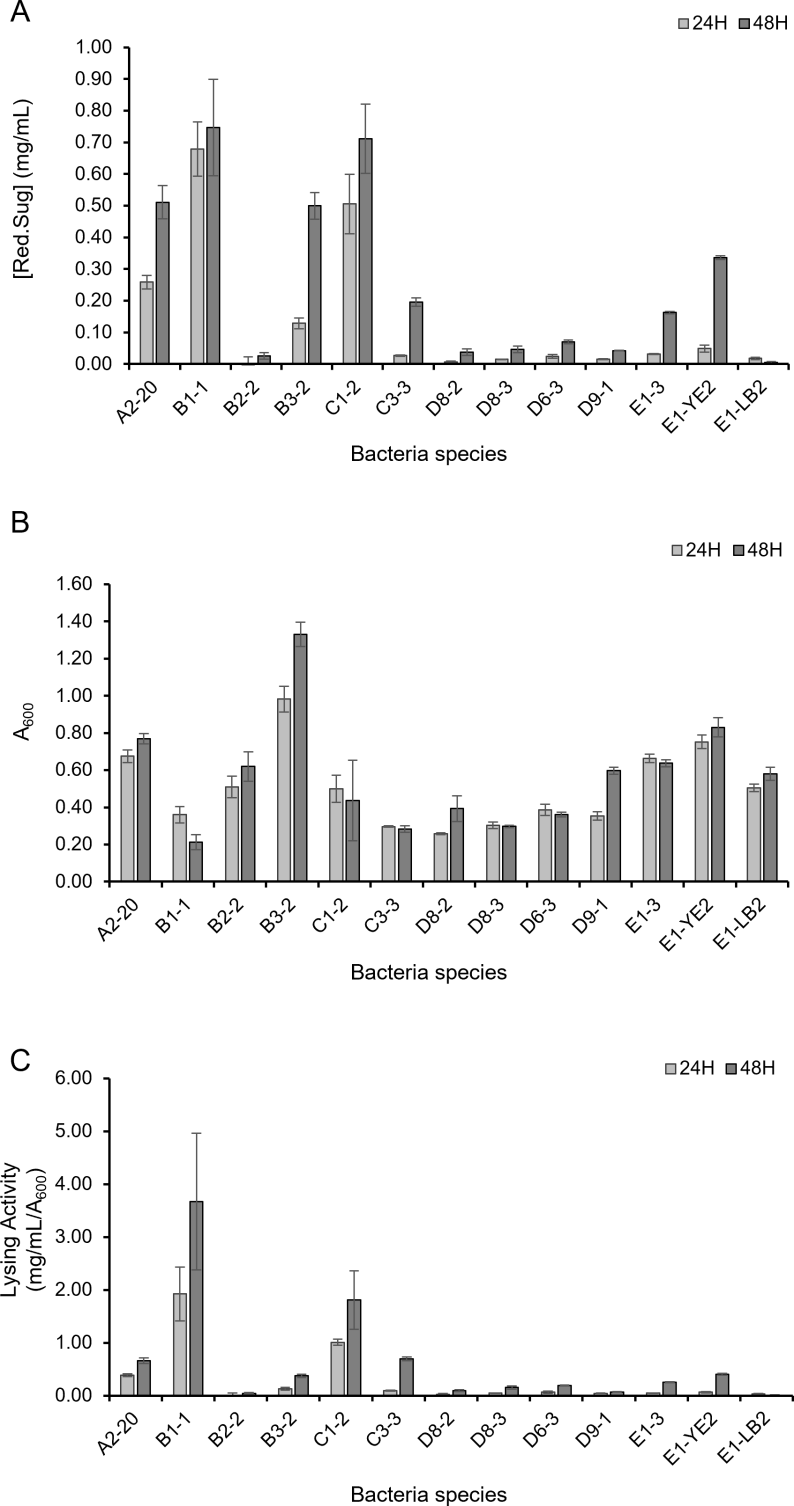
Comparison of bacterial alginate-degrading activity. Thirteen alginate-degrading bacterial strains were cultured in a liquid medium containing alginate, and the amount of reducing sugars produced was quantified using the DNS method at 24 and 48 hours. (**A**) Reducing sugar production at 24 and 48 hours was compared. (**B**) Bacterial growth at each time point was assessed by measuring absorbance. (**C**) Reducing sugar production was normalized to bacterial growth, and the resulting values were compared. All values represent the means of three independent experiments, with error bars indicating standard deviations.

To analyze the alginate-degrading activity of isolated bacteria, the amount of reducing sugar was measured after incubation in 0.4% alginate minimal medium for 24 and 48 hours (Fig. 2A). B1-1, C1-2, A2-20, B3-2, and E1-YE2 produced over 0.2 mg/mL of reducing sugar within 48 hours, suggesting that these strains exhibit higher enzyme production or enhanced enzymatic activity for alginate degradation. The bacterial growth was also measured at these two time points (Fig. 2B). A2-20, B3-2, and E1-YE2 exhibited increased growth for 24 to 48 hours, and B3-2 showed the highest growth rates among the tested bacteria. In addition, the alginate degradation activity was increased from 24 to 48 hours in the alginate minimal nutrient medium (Fig. 2A). These results suggest the possibility that these three strains, particularly B3-2, initially grew by utilizing alginate as a nutrient. While the growth of B1-1 and C1-2 decreased from 24 to 48 hours, the alginate degradation activity was significantly increased (Fig. 2A and B). These results suggest that the two strains are relatively less dependent on alginate as a carbon source and are likely to encode alginate lyases with higher enzymatic activity (Fig. 2). When the reducing sugar accumulated during 48 hours was compared, B1-1, C1-2, A2-20, B3-2, and E1-YE2 showed relatively high alginate degradation activity (Fig. 2C). When comparing the bacterial growth with the alginate-degrading activity, most strains showed active growth up to 24 hours and thereafter exhibited high alginate degradation activity until 48 hours.

### Analysis of alginate lyase secretion

Alginate lyases are generally secreted from the cell and break down alginate into monosaccharides or oligosaccharides, making them available to bacteria (48, 52). However, Cheng et al*.* reported an alginate-degrading bacterium *Cobetia* sp. cqz5-12, whose alginate lyase activity is over 15-fold higher in the intracellular fraction than in the culture supernatant (53), suggesting a need for determining intracellular activity.

**Fig 3:**
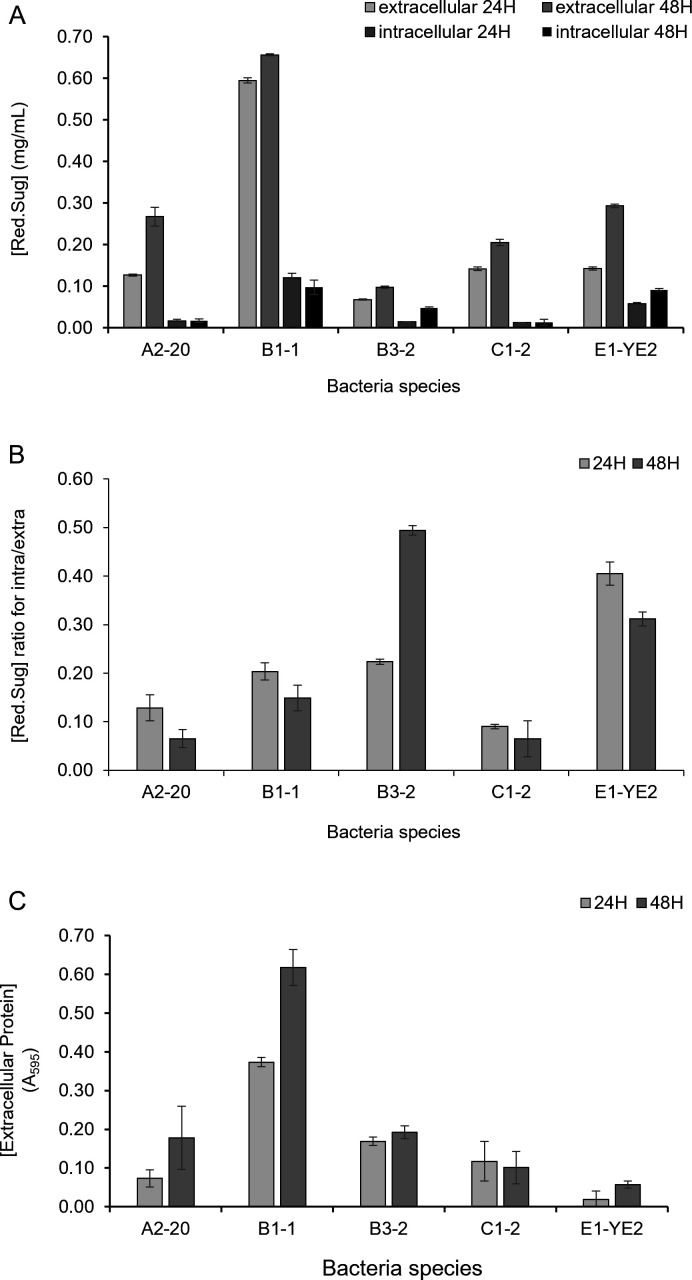
Analysis of alginate lyase secretion of alginate-degrading bacteria. To determine the cellular localization of alginate lyase activity, the reducing sugar production activity was compared between intracellular and extracellular fractions at 24 and 48 hours. (**A**) Reducing sugar production activities in intracellular and extracellular fractions were compared. (**B**) The relative intracellular alginate-degrading activity of each strain was normalized to its extracellular alginate-degrading activity and compared. (**C**) The amount of extracellular protein was quantified using the Bradford assay. All values represent the means of three independent experiments, with error bars indicating standard deviations.

Five bacteria with higher alginate degradation, A2-20, B1-1, B3-2, C1-2, and E1-YE2, were investigated to examine the localization of alginate lyase (Fig. 3A). The culture medium and cell disruption supernatant were separately studied for extracellular and intracellular alginate degradation. All the strains showed higher alginate degradation with the extracellular fraction than the intracellular fraction, indicating that alginate lyases are secreted from cells as expected. However, the relative levels of intracellular alginate degradation varied between strains (Fig. 3B). While the intracellular degradation activities of A2-20, B1-1, and C1-2 were lower than 20% of the extracellular activities, B3-2 and E1-YE2 retained 20 to 50% of the extracellular alginate lyase activity within the cells. These results suggest that microbial alginate lyases have varying secretion efficiencies or that bacteria express more than one alginate lyase with different localizations. Assuming that expression of the alginate-decomposing enzyme is preferentially induced by alginate in the minimal medium and secreted, the proteins in the culture supernatant were quantified by the Bradford assay (Fig. 3C). B1-1 displayed higher extracellular protein levels, aligning with its stronger extracellular alginate-decomposing activity than the other four strains. Despite having the lowest extracellular protein content, E1-YE2 exhibited extracellular alginate-decomposing activity comparable to A2-20 and C1-2. These results suggest that E1-YE2 may produce an alginate-decomposing enzyme with higher activity.

### Determination of alginate substrate specificity

**Fig 4:**
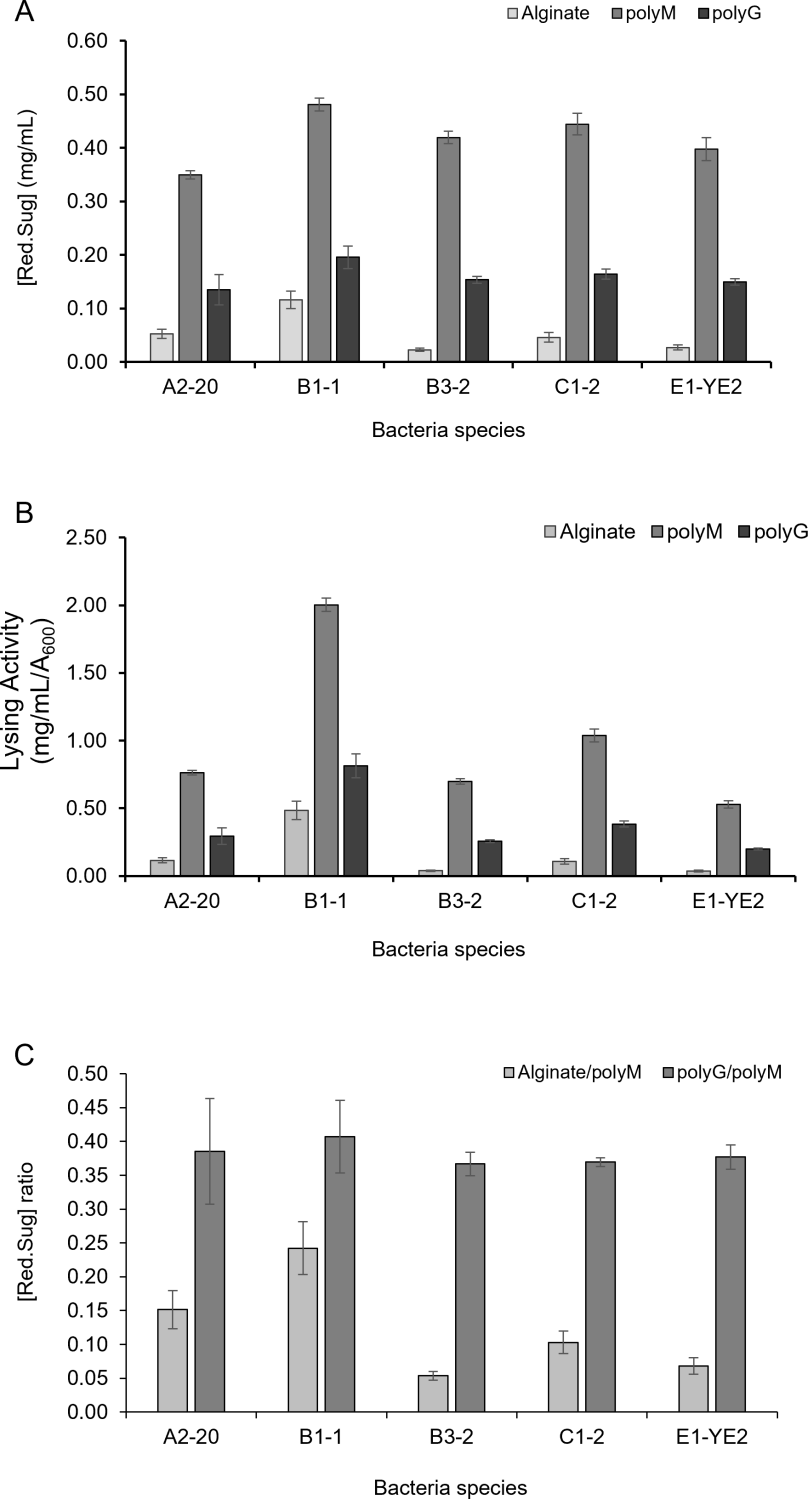
Identification of substrate specificity of alginate-degrading bacteria. The alginate-degrading activity of the isolated strains was analyzed using three different alginate substrates: alginate (heteropolymeric alginate), polymannuronate (polyM), and polyguluronate (polyG). (**A**) The amount of reducing sugars produced by the strains cultured in media containing each of the three substrates was compared. (**B**) The substrate specificity of the alginate-degrading enzymes produced by the strains was assessed by calculating the activity ratio toward heteropolymeric alginate and homopolymeric polyG relative to the activity toward homopolymeric polyM. (**C**) Alginate-degrading activity was normalized to bacterial growth by dividing the amount of reducing sugars produced by the optical density (OD) of the culture. All values represent the means of three independent experiments, with error bars indicating standard deviations.

Alginic acid is a linear unbranched polysaccharide composed of β(1→4) D-mannuronate and α(1→4) L-guluronate in an epimeric relationship. Depending on the source of algae, alginates contain homopolymeric blocks of consecutive guluronate, mannuronate, or alternating guluronate and mannuronate residues, each with different conformational preferences and behaviors. Alginate lyases are classified into polyM (poly-mannuronate) lyase, polyG (poly-guluronate) lyase, or bifunctional lyase, based on their cleavage preference (13). Three types of substrates, sodium alginate, poly-G, and poly-M, were tested to determine the substrate specificity of the isolated bacteria (Fig. 4). Five strains, A2-20, B1-1, B3-2, C1-2, and E1-YE2, exhibited the highest alginate degradation activity with poly-M. They also showed higher alginate lyase activity with poly-G than sodium alginate, indicating that they express alginate lyases that preferentially cleave homopolymeric alginate (Fig. 4A and C). A2-20 and B1-1 exhibited relatively higher degradation activity toward heteropolymeric alginate than the other three strains, B3-2, C1-2, and E1-YE2 (Fig. 4C), suggesting distinct substrate specificity of enzymes produced in these two strains. Notably, B1-1 displayed the highest lysing activity against all three substrates examined (Fig. 4B).

### Biofilm formation of alginate-degrading bacteria

**Fig 5:**
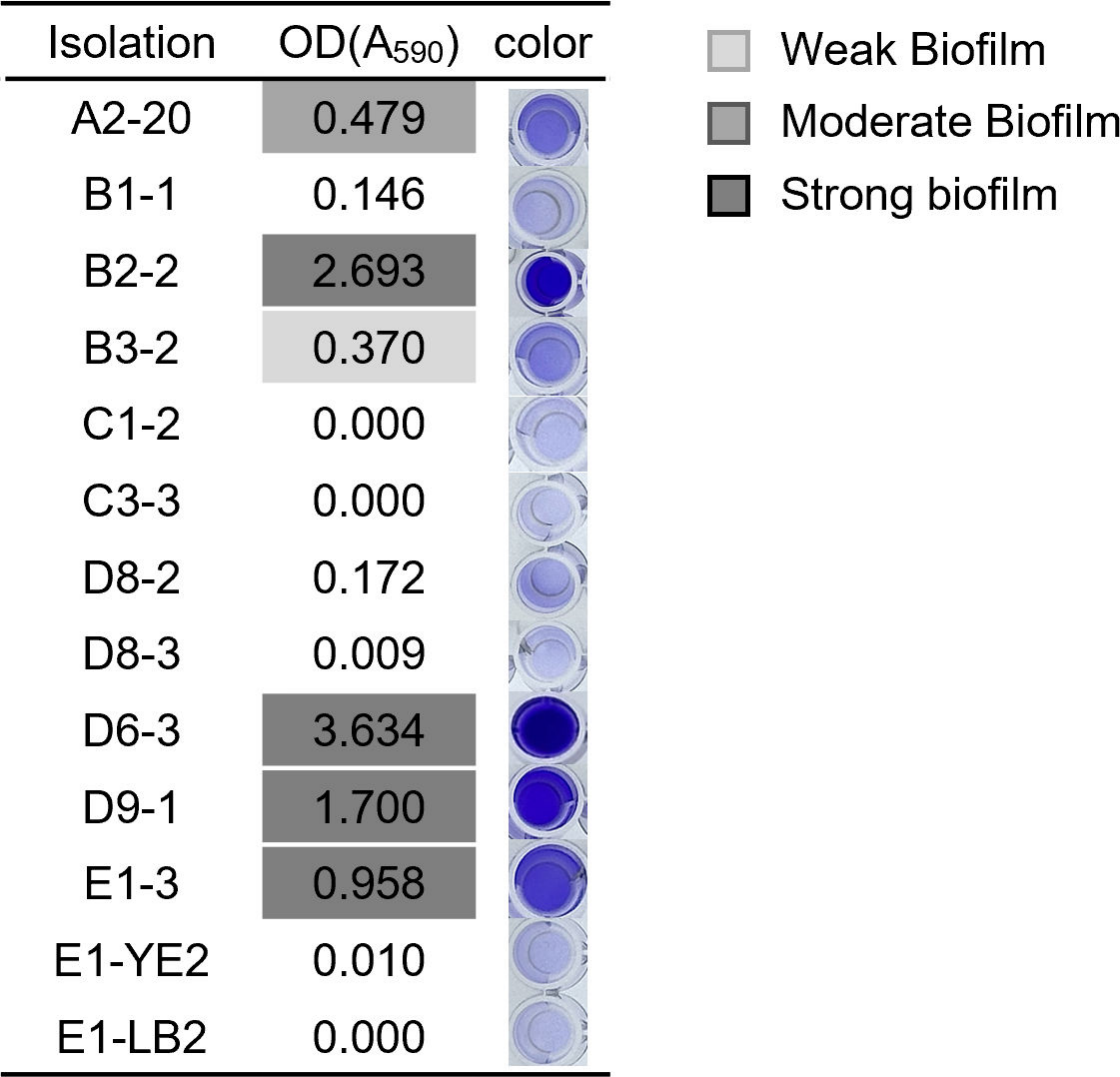
Biofilm formation by alginate-degrading bacteria. The biofilm-forming ability of the bacterial strains was assessed using a crystal violet colorimetric assay. Biofilm formation was quantified by measuring absorbance, and the results were categorized into weak, moderate, or strong biofilm formers based on the measured values. All experiments were performed in triplicate, and the average values were indicated.

Bacterial biofilms consist of microbial cells embedded in an extracellular polymeric substance (EPS) matrix containing nucleic acids, lipids, proteins, and polysaccharides like alginate. These biofilms allow bacteria to attach to plant surfaces, forming protective layers on leaves and roots that help plants withstand environmental stresses and potentially improve crop growth and yield (53, 54). Some alginate-degrading bacteria produce biofilms made of alginate to use alginate as a structural component (54, 55). They degrade biofilms for growth and dominate the soil bacterial community (56). To characterize the functional role of isolated bacteria, the biofilm formation ability of 13 strains was investigated based on colorimetric detection by crystal violet staining (Fig. 5). B2-2, D6-3, D9-1, and E1-3, which had minimal alginate degradation (Fig. 2C), showed strong biofilm formation ability. While B1-1 and C1-2, with the highest alginate-lysing activities, exhibited no to weak biofilm formation, A2-20 and B3-2 showed mild alginate degradation activities and moderate and weak biofilm formation (Fig. 2C and 5). Together, these data suggest a potential inverse correlation between alginate lyase activity and biofilm formation. In addition to alginate lyase activity, several factors, including bacterial adhesion ability, EPS composition, and nutrient availability, are known to influence biofilm formation. Further studies are needed to elucidate the precise mechanisms underlying this relationship, incorporating comprehensive transcriptomic and metabolomic analyses to investigate biofilm-associated gene expression and alginate metabolism.

### Auxin biosynthesis by alginate-degrading bacteria

**Fig 6:**
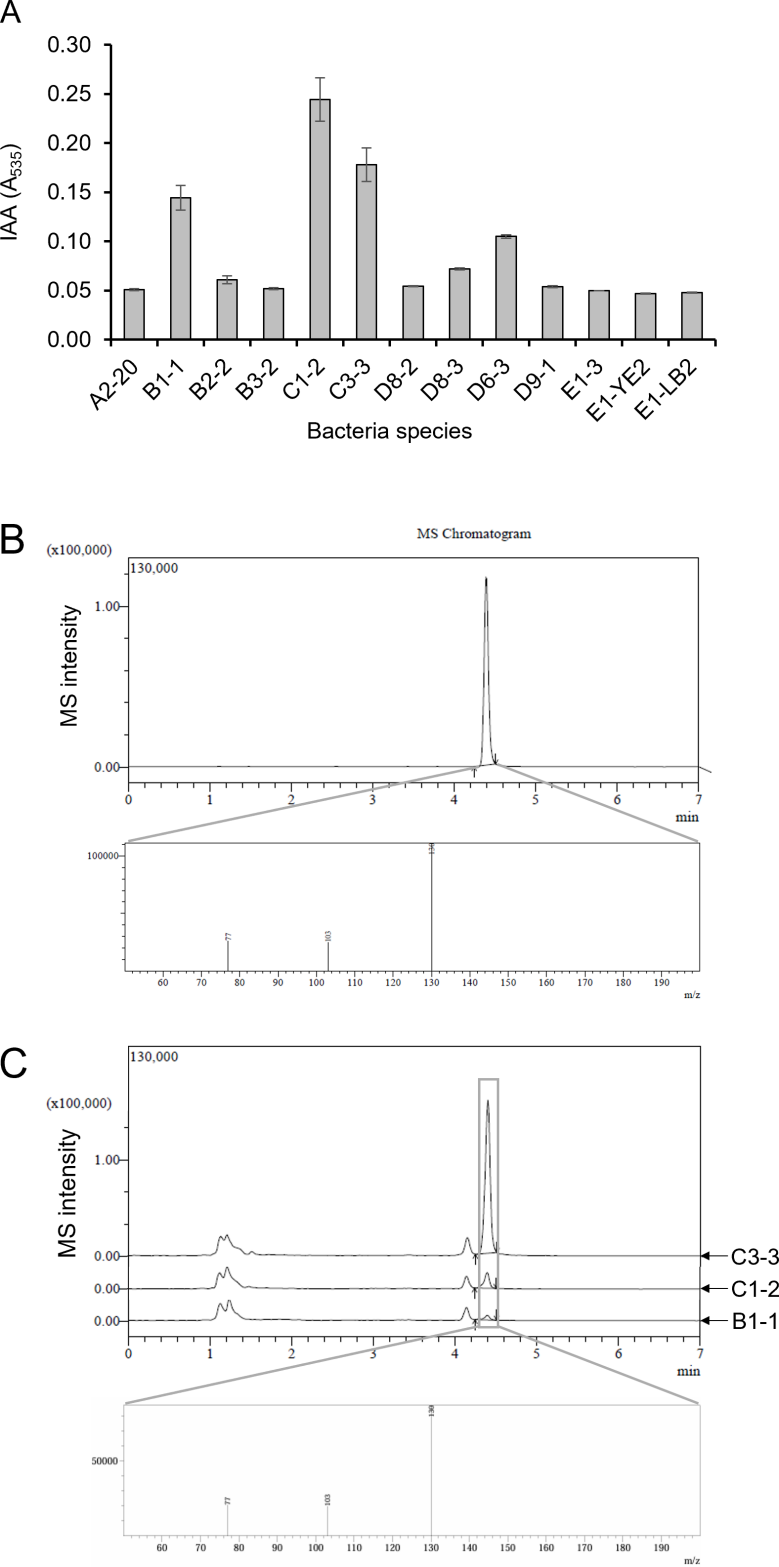
Auxin biosynthesis by alginate-degrading bacteria. The auxin production ability of the isolated bacterial strains was analyzed using chromatographic and instrumental techniques. (**A**) Auxin biosynthesis in bacterial cultures was quantified using the Salkowski assay. All values represent the means of three independent experiments, with error bars indicating standard deviations. (**B and C**) The production of IAA was confirmed and quantified through LC-MS/MS analysis of bacterial cultures. (**B**) The IAA standard’s retention time and mass spectrometry (MS) pattern were analyzed. (**C**) LC-MS/MS chromatograms and MS patterns of strains B1-1, C1-2, and C3-3 bacterial cultures are shown.

The production of auxin was examined to study the effect of the alginate-degrading bacteria on plant growth. B1-1, C1-2, and C3-3 showed stronger responses by the Salkowski test than the other strains, indicating the presence of auxins (Fig. 6A). LC-MS/MS analysis was also performed to verify and quantify the production of auxins by these bacteria. IAA, the most common naturally occurring plant hormone, was specifically targeted in this study. The standard IAA was eluted at a retention time of 4.39 minutes (Fig. 6B). The bacterial culture supernatants of B1-1, C1-2, and C3-3 had a peak with the same retention time and MS spectra as the standard IAA (Fig. 6B and C). Based on the peak area at the corresponding retention time, the total amount of IAA produced per day by each bacterium was 15.81 ppb, 50.39 ppb, and 469.04 ppb, respectively (Fig. 6C). The C3-3 strain showed the highest IAA production, approximately 9 times and 30 times higher than C1-2 and B1-1. An inconsistency was observed between the data from the Salkowski test and the LC-MS/MS analysis. One possible explanation is that B1-1 and C1-2 may produce different types of auxins, such as 4-chloro-IAA and indole-3-butyric acid (IBA), which also react with the Salkowski reagent.

### Effect of alginate-degrading bacteria on plant growth

**Fig 7:**
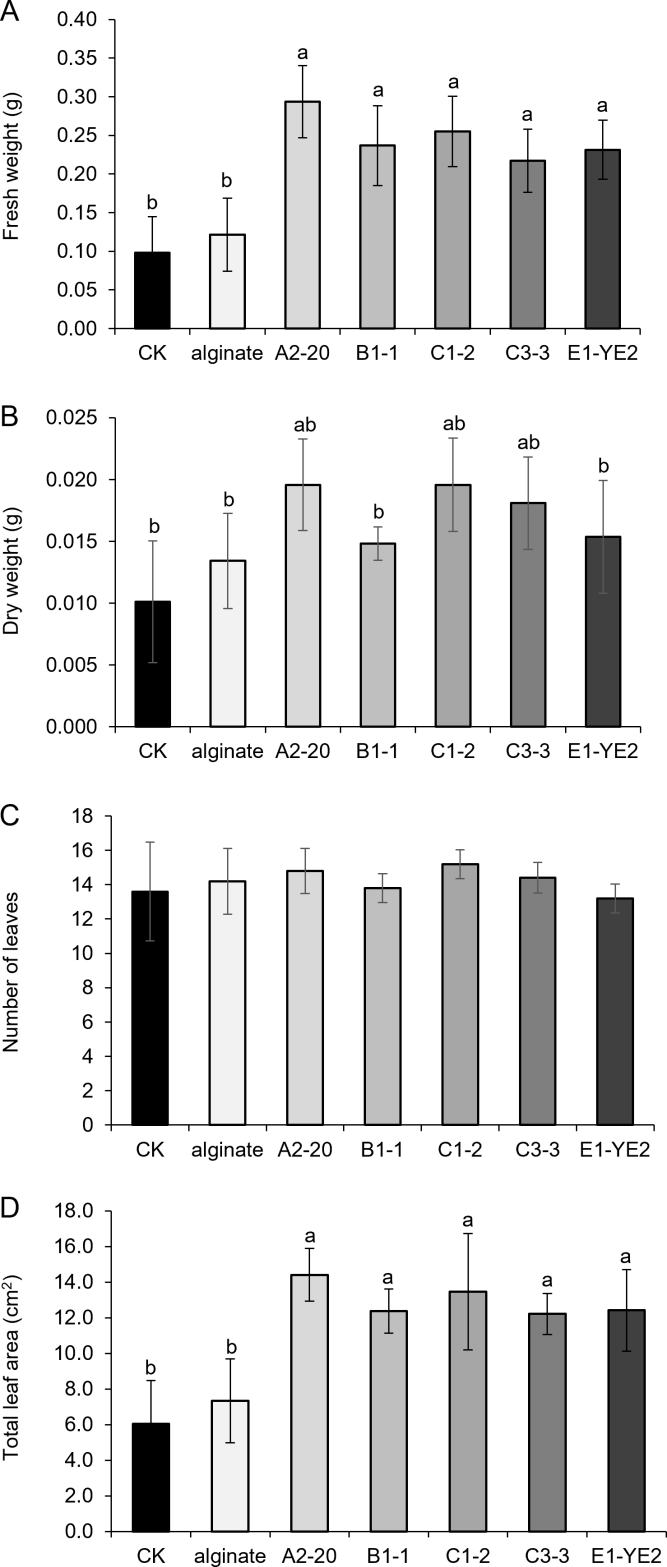
Effects of alginate-degrading bacteria on *Arabidopsis thaliana* growth. The plant growth-promoting activity of five bacterial strains was evaluated by treating the *Arabidopsis thaliana*. (**A-D**) After four bacterial treatments over 4 weeks, the fresh weight (**A**), dry weight (**B**), number of leaves (**C**), and total leaf area (**D**) of Arabidopsis were measured and compared. All values represent the means of five independent experiments, with error bars indicating standard deviations. Significant differences among treatments were analyzed using Tukey’s Honest Significant Difference (HSD) test (*P* < 0.05).

A2-20, B1-1, C1-2, C3-3, and E1-YE2 strains, which showed relatively higher alginate-degrading activities (Fig. 2B and C), were investigated for their impact on plant growth. Among these strains, B1-1, C1-2, and C3-3 were demonstrated to produce auxins, particularly IAA (Fig. 6). *Arabidopsis thaliana* (Col-0) was treated with the bacterial cells resuspended in 0.4% (wt/vol) alginate solution weekly for 5 weeks, and the growth of *A. thaliana* was monitored (Fig. S2 and Table S2). While the alginate solution without bacterial cells did not affect plant growth, treating bacterial cells led to the overall enhancement in the weight of leaves and/or total leaf area (Fig. 7). Plants treated with B1-1, C1-2, and C3-3, which have both alginate degradation activity and IAA biosynthesis ability, significantly increased the fresh weight and total leaf area compared to the control (Fig. 7A and D). In addition, A2-20 and E1-YE2, with only alginate-degrading activity, showed significant growth promotion effects regarding fresh weight and total leaf area. These findings support that bacterial alginate-degrading activity promotes plant growth, regardless of auxin production. Alginate oligosaccharides (AOSs) were shown to promote the growth of *Arabidopsis thaliana* (23, 54) and also induce the expression of auxin-related genes (54). Therefore, it was difficult to clearly distinguish the individual roles of auxin and AOSs in promoting the vegetative growth of plants. Further research is required to elucidate the mechanisms underlying plant growth promotion by alginate-degrading bacteria and determine the effect of AOS-mediated auxin-related gene expression on plant growth.

## DISCUSSION

Studies on bacteria capable of degrading alginate, a key cell wall component of brown algae, have been conducted since 1954 (55). Alginate oligosaccharides (AOSs) are non-immunogenic, non-toxic, and biodegradable, making them highly valuable for food, pharmaceutical, and bioenergy applications. Among the various processing methods to produce AOS (56–60), enzymatic degradation of alginate by alginate lyase is favored due to its stability, eco-friendliness, and environmental sustainability (61). Alginate lyase breaks down alginate into AOS ranging from 2 to 25 monomers through β-elimination (13, 62). AOS is known to exhibit a range of physiological functions. In particular, the tri-saccharides AOS were reported to regulate plant growth and enhance root development in agricultural applications (3, 25, 63–66). The biological function of AOSs is influenced by various factors, including their molecular weight, degree of polymerization, M/G ratio, and terminal saturation. AOSs produced by bacterial alginate lyases have been studied extensively for application in various fields (65).

In this study, 13 bacterial strains were isolated from the coastal area of South Korea, and the properties of alginate-degrading activity were characterized. Previous studies have suggested the potential alginate-degrading ability of *Vibrio* bacteria. *Vibrio* sp. C8-15, isolated from Linnaeus (*Atrina pectinata*), showed a reducing sugar production activity of 1.07 mg/mL within 6 hours (67), and *Vibrio* sp. PKA 1003, isolated from seaweed, produced 0.740 mg/mL of reducing sugars in 12 hours (68). In our study, *Vibrio* sp. exhibited the highest alginate-degrading activity, highlighting their potential for industrial applications. Three *Vibrio* sp. strains, *Vibrio* sp. strain B1-1, *Vibrio* sp. strain C1-2, and *Vibrio* sp. strain B3-2, produced reducing sugars of 0.747 mg/mL, 0.711 mg/mL, and 0.499 mg/mL, respectively, after 48 hours of incubation. This study is the first to report the alginate-degrading capability of the *Zobellella* genus, highlighting the need for further investigation into its alginate decomposition mechanisms and potential for commercial applications.

Six isolates were identified as biofilm-forming bacteria. Biofilm-forming plant growth-promoting bacteria (PGPB) are known to contribute to improved soil microbial balance and enhanced crop productivity (53, 69, 70). For instance, certain strains of *Pseudomonas sp*. and *Bacillus sp*. have been reported to produce polysaccharide-based biofilms, including those composed of alginate (71–74). In addition, *Pseudomonas aeruginosa* and *Helicobacter pylori* have been shown to utilize alginate-degrading activity during biofilm formation and maturation processes (69, 70, 75). An experimental analysis of the relationship between alginate-degrading activity and biofilm formation revealed a potential inverse correlation between these traits. As biofilm formation is influenced by various factors, including bacterial adhesion, EPS composition, and environmental conditions, further studies are required to elucidate the specific effects of alginate lyase activity on biofilm formation.

The IAA biosynthesis is one of the key characteristics of PGPB. Species such as *Bacillus sp*. and *Rhizobium sp*. are known to produce IAA, which plays crucial roles in regulating embryo development, leaf expansion, phototropism, and geotropism in crops (76–79) while also promoting root growth to enhance nutrient uptake (80, 81). In addition, Zhang et al. reported that AOS produced by alginate-degrading bacteria can induce auxin biosynthesis and exert plant growth-promoting effects (54). In this study, *Vibrio* sp. strain C1-2 and *Marinomonas* sp. strain C3-3 possess alginate-degrading activity and auxin-producing ability. These strains significantly promoted the growth of *Arabidopsis thaliana*, further supporting their potential roles in plant-microbe interactions.

Interestingly, *Stenotrophomonas* sp. strain A2-20, which exhibits alginate-degrading activity and biofilm-forming ability, also demonstrated plant growth-promoting effects. Notably, these isolated bacteria did not exhibit auxin-producing capability, suggesting that their plant growth-promoting effects occur independently of auxin production. This may be attributed to the beneficial effects of AOS on plant growth. However, the mechanisms responsible for the plant growth-promoting effects of *Stenotrophomonas* are not yet fully understood. Future studies should focus on elucidating these mechanisms through integrated transcriptomic, metabolomic, and *in situ* soil microbiome analyses to facilitate the practical application of these strains in agricultural systems. Leveraging the potential of these PGPB could contribute to developing environmentally sustainable farming strategies that reduce reliance on chemical fertilizers and promote long-term crop productivity.

## Data Availability

The data underlying this article are available in the article and its supplemental material.

## SUPPLEMENTAL MATERIAL

Supplemental figures and tables spectrum.03164-24-s0001.docx The standard curve and reaction result plate for the DNS assay, 16S rDNA sequences of isolated bacteria, *Arabidopsis thaliana* growth promotion results, and a one-way ANOVA table.

ASM does not own the copyrights to Supplemental Material that may be linked to, or accessed through, an article. The authors have granted ASM a non-exclusive, world-wide license to publish the Supplemental Material files. Please contact the corresponding author directly for reuse.
