# Supplementary material for: Characterization of alginate-degrading bacteria isolated from seaweed-producing areas of South Korean territory and verification of the bacteria as plant growth-promoting biofertilizer
Source: Microbiol Spectr. 2025 Apr 29;13(6):e03164-24. doi: 10.1128/spectrum.03164-24 (PMC12131846; doi:10.1128/spectrum.03164-24)
Supplement: Supplemental figures and tables — The standard curve and reaction result plate for the DNS assay, 16S rDNA sequences of isolated bacteria, Arabidopsis thaliana growth promotion results, and a one-way ANOVA table. [file spectrum.03164-24-s0001.docx]

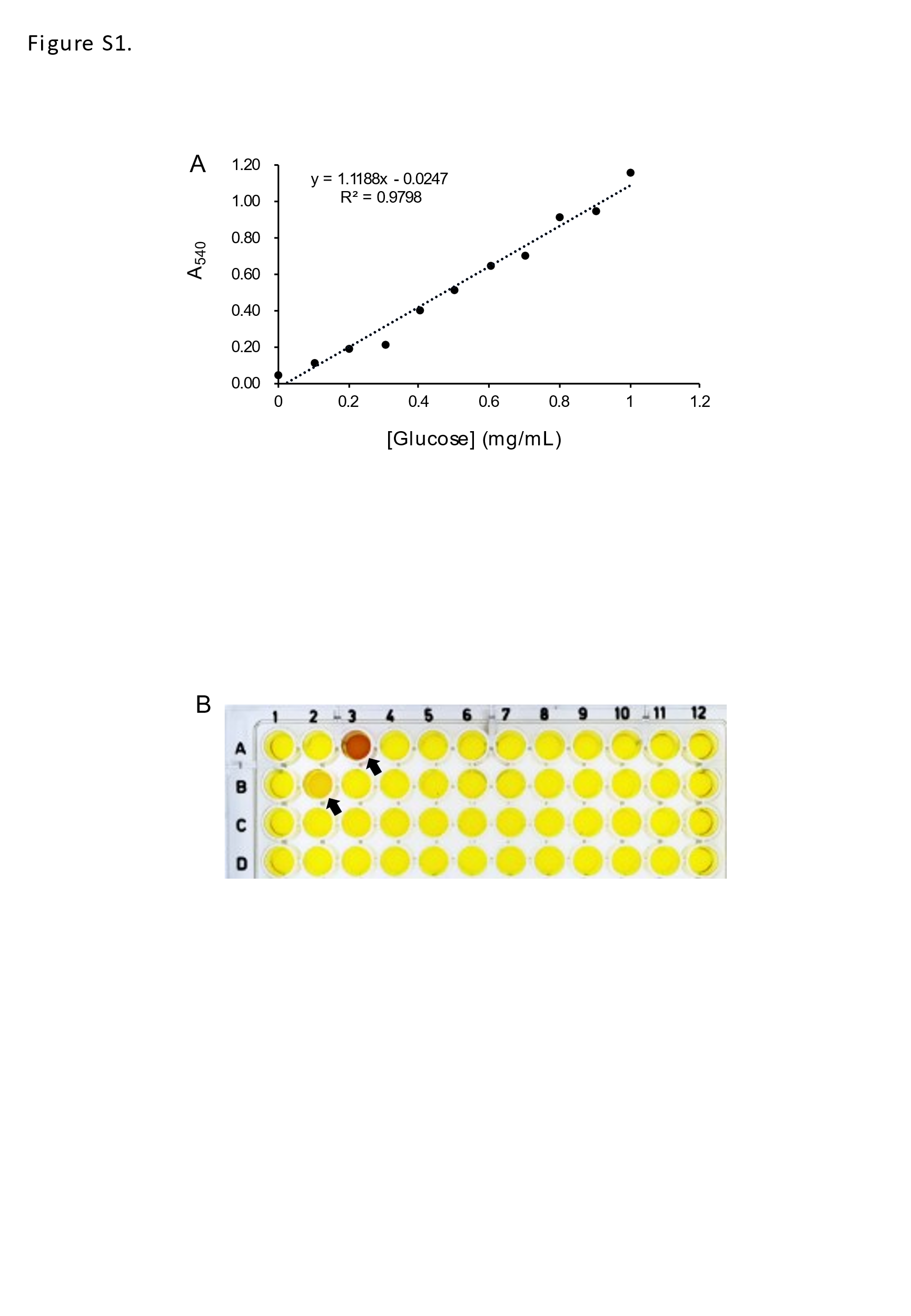
**Figure S1.**

**Supplementary Fig 1.** Application of the DNS assay for reducing sugars in screening alginate-degrading bacteria. (A) A standard curve illustrating the relationship between DNS color development and reducing sugar concentration. (B) Detection of alginate-degrading bacteria based on distinct color differences observed in a 96-well plate assay.

**Figure S2.**

**
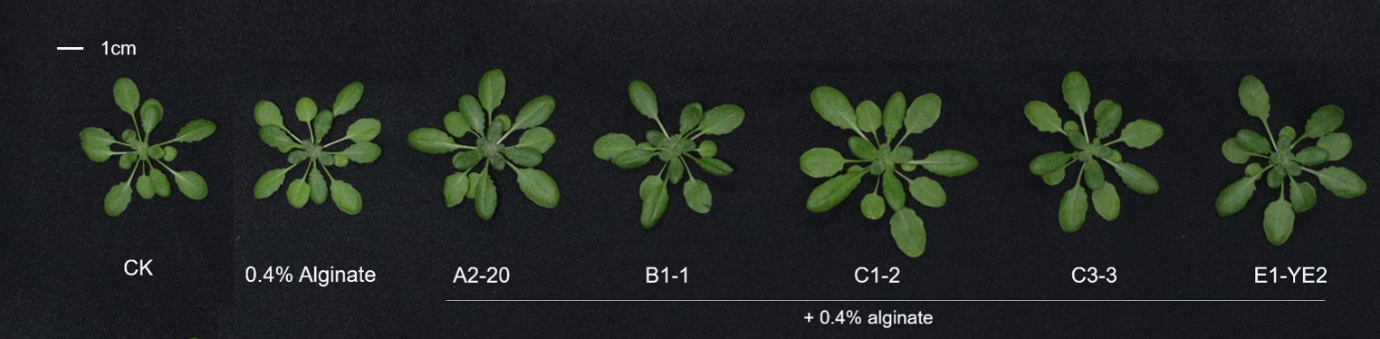
**

**Supplementary Fig 2.** Observation of the effects of alginate and alginate-degrading bacteria on plant growth promotion. Bacterial strains exhibiting alginate-degrading activity without IAA-biosynthetic capability (A2-20, E1-YE2) and strains possessing both alginate-degrading activity and IAA biosynthetic capability (B1-1, C1-2, C3-3) were applied for four weeks, and their effects on plant growth were evaluated.

**Table S1.** 16S rDNA gene sequence of isolated alginate-degrading bacteria.

| **Isolation** | | **Identification** | **16s rDNA sequence** |
| --- | --- | --- | --- |
| A2-20 | | *Stenotrophomonas*  sp. A2-20 | ACACATGCAGTCGAACGGCAGCACAGGAGAGCTTGCTCTCTGGGTGGCGAGTGGCGGACGGGTGAGGAATACATCGGAATCTACTTTTTCGTGGGGGATAACGTAGGGAAACTTACGCTAATACCGCATACGACCTACGGGTGAAAGCAGGGGACCTTCGGGCCTTGCGCGATTGAATGAGCCGATATCGGATTAGCTAGTTGGCGGGGTAAAGGCCCACCAAGGCGACGATCCGTAGCTGGTCTGAGAGGATGATCAGCCACACTGGAACTGAGACACGGTCCAGACTCCTACGGGAGGCAGCAGTGGGGAATATTGGACAATGGGCGCAAGCCTGATCCAGCCATACCGCGTGGGTGAAGAAGGCCTTCGGGTTGTAAAGCCCTTTTGTTGGGAAAGAAATCCAGCCGGCTAATACCTGGTTGGGATGACGGTACCCAAAGAATAAGCACCGGCTAACTTCGTGCCAGCAGCCGCGGTAATACGAAGGGTGCAAGCGTTACTCGGAATTACTGGGCGTAAAGCGTGCGTAGGTGGTTGTTTAAGTCTGTTGTGAAAGCCCTGGGCTCAACCTGGGAACTGCAGTGGAAACTGGACGACTAGAGTGTGGTAGAGGGTAGCGGAATTCCTGGTGTAGCAGTGAAATGCGTAGAGATCAGGAGGAACATCCATGGCGAAGGCAGCTACCTGGACCAACACTGACACTGAGGCACGAAAGCGTGGGGAGCAAACAGGATTAGATACCCTGGTAGTCCACGCCCTAAACGATGCGAACTGGATGTTGGGTGCAATTTGGCACGCAGTATCGAAGCTAACGCGTTAAGTTCGCCGCCGGGGGAGTACGGTCGCAAGACTGAAACTCATAGGAATTGACGGGGGCCCGCACAAGCGGTGGAGTATGTGGTTTAATTCGATGCAACGCGAAGAACCTTACCTGGCCTTGACATGTCGAGAACTTTCCAGAGATGGATTGGTGCCTTCGGGAACTCGAACACAGGTGCTGCAGGGCTGTCGTCAGCTCGTGTCGTGAGATGTTGGGTTAAGTCCCGCAACGAGCGCAACCCTTGTCCTTAGTTGCCAGCACGTAATGGTGGGAACTCTAAGGAGACCGCCGGTGACAAACCGGAGGAAGGTGGGGATGACGTCAAGTCATCATGGCCCTTACGGCCAGGGCTACACACGTACTACAATGGTAGGGACAGAGGGCTGCAAGCCGGCGACGGTAAGCCAATCCCAGAAACCCTATCTCAGTCCGGATTGGAGTCTGCAACTCGACTCCATGAAGTTGGAATCGCTAGTAATCGCAGATCAGCATTGCTGCGGTGAATACGTTCCCGGGCCTTGTACACACCGCCCGTCACACCATGGGAGTTTGTTGCACCAGAAGCAGGTAGCTTAACCTTCGGGAGGGCGCTGCCA |
| B1-1 | | *Vibrio* sp. B1-1 | TGCAAGTCGAGCGGAACGAGAATAGCTTGCTATTCGGCGTCGAGCGGCGGACGGGTGAGTAATGCATAGGAAGTTGCCCAGTAGAGGGGGATAACCATTGGAAACGATGGCTAATACCGCATAACCTCTTTGGAGCAAAGCGGGGGACCTTCGGGCCTCGCGCTACTGGATACGCCTATGTGGGATTAGCTAGTTGGTGAGGTAAAGGCTCACCAAGGCGACGATCCCTAGCTGGTCTGAGAGGATGATCAGCCACACTGGGACTGAGACACGGCCCAGACTCCTACGGGAGGCAGCAGTGGGGAATATTGCACAATGGGCGAAAGCCTGATGCAGCCATGCCGCGTGTATGAAGAAGGCCTTCGGGTTGTAAAGTACTTTCAGTTGTGAGGAAGGTTTCGTAGTTAATAACTGCGTTGCTTGACGTTAGCAACAGAAGAAGCACCGGCTAACTCCGTGCCAGCAGCCGCGGTAATACGGAGGGTGCGAGCGTTAATCGGAATTACTGGGCGTAAAGCGCATGCAGGTGGTTTGTTAAGTCAGATGTGAAAGCCCGGGGCTCAACCTCGGAAGGTCATTTGAAACTGGCAAACTAGAGTACTGTAGAGGGGGGTAGAATTTCAGGTGTAGCGGTGAAATGCGTAGAGATCTGAAGGAATACCAGTGGCGAAGGCGGCCCCCTGGACAGATACTGACACTCAGATGCGAAAGCGTGGGGAGCAAACAGGATTAGATACCCTGGTAGTCCACGCCGTAAACGATGTCTACTTGGAGGTTGTGGCCTTGAGCCGTGGCTTTCGGAGCTAACGCGTTAAGTAGACCGCCTGGGGAGTACGGTCGCAAGATTAAAACTCAAATGAATTGACGGGGGCCCGCACAAGCGGTGGAGCATGTGGTTTAATTCGATGCAACGCGAAGAACCTTACCTACTCTTGACATCCAGAGAACTTTCCAGAGATGGATTGGTGCCTTCGGGAACTCTGAGACAGGTGCTGCATGGCTGTCGTCAGCTCGTGTTGTGAAATGTTGGGTTAAGTCCCGCAACGAGCGCAACCCTTATCCTTGTTTGCCAGCGAGTAATGTCGGGAACTCCAGGGAGACTGCCGGTGATAAACCGGAGGAAGGTGGGGACGACGTCAAGTCATCATGGCCCTTACGAGTAGGGCTACACACGTGCTACAATGGCGCATACAGAGGGCAGCGAACTTGCGAAAGTAAGCGAATCCCAAAAAGTGCGTCGTAGTCCGGATTGGAGTCTGCAACTCGACTCCATGAAGTCGGAATCGCTAGTAATCGTGGATCAGAATGCCACGGTGAATACGTTCCCGGGCCTTGTACACACCGCCCGTCACACCATGGGAGTGGGCTGCAAAAGAAGTAGGTAGTTTAACCTTCGGGAGGACGCTAC |
| B2-2 | | *Bacillus* sp. B2-2 | TGCAAGTCGAGCGAATGGATTGAGAGCTTGCTCCTCAAGAAGTTAGCGGCGGACGGGTGAGTAACACGTGGGTAACCTGCCCATAAGACTGGGATAACTCCGGGAAACCGGGGCTAATACCGGATAACATTTTGAACTGCATGGTTCGAAATTGAAAGGCGGCTTCGGCTGTCACTTATGGATGGACCCGCGTCGCATTAGCTAGTTGGTGAGGTAACGGCTCACCAAGGCAACGATGCGTAGCCGACCTGAGAGGGTGATCGGCCACACTGGGACTGAGACACGGCCCAGACTCCTACGGGAGGCAGCAGTAGGGAATCTTCCGCAATGGACGAAAGTCTGACGGAGCAACGCCGCGTGAGTGATGAAGGCTTTCGGGTCGTAAAACTCTGTTGTTAGGGAAGAACAAGTGCTAGTTGAATAAGCTGGCACCTTGACGGTACCTAACCAGAAAGCCACGGCTAACTACGTGCCAGCAGCCGCGGTAATACGTAGGTGGCAAGCGTTATCCGGAATTATTGGGCGTAAAGCGCGCGCAGGTGGTTTCTTAAGTCTGATGTGAAAGCCCACGGCTCAACCGTGGAGGGTCATTGGAAACTGGGAGACTTGAGTGCAGAAGAGGAAAGTGGAATTCCATGTGTAGCGGTGAAATGCGTAGAGATATGGAGGAACACCAGTGGCGAAGGCGACTTTCTGGTCTGTAACTGACACTGAGGCGCGAAAGCGTGGGGAGCAAACAGGATTAGATACCCTGGTAGTCCACGCCGTAAACGATGAGTGCTAAGTGTTAGAGGGTTTCCGCCCTTTAGTGCTGAAGTTAACGCATTAAGCACTCCGCCTGGGGAGTACGGCCGCAAGGCTGAAACTCAAAGGAATTGACGGGGGCCCGCACAAGCGGTGGAGCATGTGGTTTAATTCGAAGCAACGCGAAGAACCTTACCAGGTCTTGACATCCTCTGAAAACCCTAGAGATAGGGCTTCTCCTTCGGGAGCAGAGTGACAGGTGGTGCATGGTTGTCGTCAGCTCGTGTCGTGAGATGTTGGGTTAAGTCCCGCAACGAGCGCAACCCTTGATCTTAGTTGCCATCATTAAGTTGGGCACTCTAAGGTGACTGCCGGTGACAAACCGGAGGAAGGTGGGGATGACGTCAAATCATCATGCCCCTTATGACCTGGGCTACACACGTGCTACAATGGACGGTACAAAGAGCTGCAAGACCGCGAGGTGGAGCTAATCTCATAAAACCGTTCTCAGTTCGGATTGTAGGCTGCAACTCGCCTACATGAAGCTGGAATCGCTAGTAATCGCGGATCAGCATGCCGCGGTGAATACGTTCCCGGGCCTTGTACACACCGCCCGTCACACCACGAGAGTTTGTAACACCCGGAAGTCGGTGGGGTAACCTTTATGGAGCCAGCCG |
| B3-2 | | *Vibrio* sp. B3-2 | ATGCAGTCGAGCGGAACGAGTTATCTGAACCTTCGGGGGACGATACGGCGTCGAGCGGCGGACGGGTGAGTAATGCCTAGGAAATTGCCCTGATGTGGGGGATAACCATTGGAAACGATGGCTAATACCGCAGATGCCTACGGGCCAAAGAGGGGGACCTTCGGGCCTCTCGCGTCAGGATATGCCTAGGTGGGATTAGCTAGTTGGTGAGGTAAGGGCTCACCAAGGCGACGATCCCTAGCTGGTCTGAGAGGATGATCAGCCACACTGGAACTGAGACACGGTCCAGACTCCTACGGGAGGCAGCAGTGGGGAATATTGCACAATGGGCGCAAGCCTGATGCAGCCATGCCGCGTGTGTGAAGAAGGCCTTCGGGTTGTAAAGCACTTTCAGTCGTGAGGAAGGTGGTGTAGTTAATAGCTGCATTATTTGACGTTAGCGACAGAAGAAGCACCGGCTAACTCCGTGCCAGCAGCCGCGGTAATACGGAGGGTGCGAGCGTTAATCGGAATTACTGGGCGTAAAGCGCATGCAGGTGGTTTGTTAAGTCAGATGTGAAAGCCCGGGGCTCAACCTCGGAATAGCATTTGAAACTGGCAGACTAGAGTACTGTAGAGGGGGGTAGAATTTCAGGTGTAGCGGTGAAATGCGTAGAGATCTGAAGGAATACCGGTGGCGAAGGCGGCCCCCTGGACAGATACTGACACTCAGATGCGAAAGCGTGGGGAGCAAACAGGATTAGATACCCTGGTAGTCCACGCCGTAAACGATGTCTACTTGGAGGTTGTGGCCTTGAGCCGTGGCTTTCGGAGCTAACGCGTTAAGTAGACCGCCTGGGGAGTACGGTCGCAAGATTAAAACTCAAATGAATTGACGGGGGCCCGCACAAGCGGTGGAGCATGTGGTTTAATTCGATGCAACGCGAAGAACCTTACCTACTCTTGACATCCAGAGAACTTTCCAGAGATGGATTGGTGCCTTCGGGAACTCTGAGACAGGTGCTGCATGGCTGTCGTCAGCTCGTGTTGTGAAATGTTGGGTTAAGTCCCGCAACGAGCGCAACCCTTATCCTTGTTTGCCAGCGAGTAATGTCGGGAACTCCAGGGAGACTGCCGGTGATAAACCGGAGGAAGGTGGGGACGACGTCAAGTCATCATGGCCCTTACGAGTAGGGCTACACACGTGCTACAATGGCGCATACAGAGGGCGGCCAACTTGCGAAAGTGAGCGAATCCCAAAAAGTGCGTCGTAGTCCGGATTGGAGTCTGCAACTCGACTCCATGAAGTCGGAATCGCTAGTAATCGTGGATCAGAATGCCACGGTGAATACGTTCCCGGGCCTTGTACACACCGCCCGTCACACCATGGGAGTGGGCTGCAAAAGAAGTAGGTAGTTTAACCTTCGGGGGGACGCTAC |
| C1-2 | *Vibrio* sp. C1-2 | | TACACATGCAGTCGAGCGGAACGAGTTAACTGAACCTTCGGGGGACGTTAACGGCGTCGAGCGGCGGACGGGTGAGTAATGCCTAGGAAATTGCCCTGATGTGGGGGATAACCATTGGAAACGATGGCTAATACCGCATGATGCCTACGGGCCAAAGAGGGGGACCTTCGGGCCTCTCGCGTCAGGATATGCCTAGGTGGGATTAGCTAGTTGGTGAGGTAAGGGCTCACCAAGGCGACGATCCCTAGCTGGTCTGAGAGGATGATCAGCCACACTGGAACTGAGACACGGTCCAGACTCCTACGGGAGGCAGCAGTGGGGAATATTGCACAATGGGCGCAAGCCTGATGCAGCCATGCCGCGTGTGTGAAGAAGGCCTTCGGGTTGTAAAGCACTTTCAGTCGTGAGGAAGGTTTATGCGTTAATAGCGTATAGATTTGACGTTAGCGACAGAAGAAGCACCGGCTAACTCCGTGCCAGCAGCCGCGGTAATACGGAGGGTGCGAGCGTTAATCGGAATTACTGGGCGTAAAGCGCATGCAGGTGGTTTGTTAAGTCAGATGTGAAAGCCCGGGGCTCAACCTCGGAATAGCATTTGAAACTGGCAGACTAGAGTACTGTAGAGGGGGGTAGAATTTCAGGTGTAGCGGTGAAATGCGTAGAGATCTGAAGGAATACCGGTGGCGAAGGCGGCCCCCTGGACAGATACTGACACTCAGATGCGAAAGCGTGGGGAGCAAACAGGATTAGATACCCTGGTAGTCCACGCCGTAAACGATGTCTACTTGGAGGTTGTGGCCTTGAGCCGTGGCTTTCGGAGCTAACGCGTTAAGTAGACCGCCTGGGGAGTACGGTCGCAAGATTAAAACTCAAATGAATTGACGGGGGCCCGCACAAGCGGTGGAGCATGTGGTTTAATTCGATGCAACGCGAAGAACCTTACCTACTCTTGACATCCAGAGAACTTTCCAGAGATGGATTGGTGCCTTCGGGAACTCTGAGACAGGTGCTGCATGGCTGTCGTCAGCTCGTGTTGTGAAATGTTGGGTTAAGTCCCGCAACGAGCGCAACCCTTATCCTTGTTTGCCAGCGAGTAATGTCGGGAACTCCAGGGAGACTGCCGGTGATAAACCGGAGGAAGGTGGGGACGACGTCAAGTCATCATGGCCCTTACGAGTAGGGCTACACACGTGCTACAATGGCGCATACAGAGGGCGGCCAACTTGCGAAAGTGAGCGAATCCCAAAAAGTGCGTCGTAGTCCGGATTGGAGTCTGCAACTCGACTCCATGAAGTCGGAATCGCTAGTAATCGTGGATCAGAATGCCACGGTGAATACGTTCCCGGGCCTTGTACACACCGCCCGTCACACCATGGGAGTGGGCTGCAAAAGAAGTAGGTAGTTTAACCTTCGGGGGGACGCTACC |
| C3-3 | *Marinomonas* sp. C3-3 | | CTTACCATGCAGTCGAGCGGTAACAGGGGAGCTTGCTCCTGCTGACGAGCGGCGGACGGGTGAGTAACGCGTAGGAATCCTACCTAGTAGAGGGGGACAACATGTGGAAACGCATGCTAATACCGCAACACCCTAAGGGGGAAAGGAGGGGACTCTTCGGAGCCTTCCGCTATTAGATGAGCCTGCGTGAGATTAGCTAGTTGGTAGGGTAAAGGCCTACCAAGGCGACGATCTCTAACTGGTCTGAGAGGATGACCAGTCACACTGGGACTGAGACACGGCCCAGACTCCTACGGGAGGCAGCAGTGGGGAATATTGGACAATGGGCGGAAGCCTGATCCAGCCATGCCGCGTGTGTGAAGAAGGCCTTAGGGTTGTAAAGCACTTTCAGGGGTGAGGAAGGGTGACTGGCTAATATCCAGTTATCTTGACGTTAGCCCCAGAAGAAGCACCGGCTAACTCTGTGCCAGCAGCCGCGGTAATACAGAGGGTGCAAGCGTTAATCGGAATTACTGGGCGTAAAGCGCGCGTAGGTGGTTTGTTAAGTCGGATGTGAAATCCCAGGGCTCAACCTTGGAATGGCACCCGATACTGGCTAGCTAGAGTATGGTAGAGGGGTGTGGAATTTCCTGTGTAGCGGTGAAATGCGTAGATTTAGGAAGGAACATCAGTGGCGAAAGCGACACCCTGGACTAATACTGACACTGAGGTGCGAAAGCGTGGGGAGCAAACAGGATTAGATACCCTGGTAGTCCACGCCGTAAACGATGTCTACTAGCCGTTGGGTTGTAATGACTTAGTGGCGCAGCTAACGCAATAAGTAGACCGCCGGGGGAGTACGGCCGCAAGGTTAAAACTCAAATGAATTGACGGGGGCCCGCACAAGCGGTGGAGCATGTGGTTTAATTCGACGCAACGCGGAGAACCTTACCTACTCTTGACATCCAGTGAATCCCAAAGAGATATTGGAGTGCCTTCGGGAACACTGAGACAGGTGCTGCATGGCTGTCGTCAGCTCGTGTTGTGAAATGTTGGGTTAAGTCCCGTAACGAGCGCAACCCTTATCCTTATTTGCCAGCACTTCGGGTGGGAACTTTAAGGAGACTGCCGGTGACAAACCGGAGGAAGGTGGGGACGACGTCAAGTCATCATGGCCCTTACGAGTAGGGCTACACACGTGCTACAATGGCGTATACAGAGGGCTGCAAGCTAGCGATAGTGAGCGAATCCCACAAAGTACGTCGTAGTCCGGATTGGAGTCTGCAACTCGACTCCATGAAGTCGGAATCGCTAGTAATCGTGGATCAGAATGCCACGGTGAATACGTTCCCGGGCCTTGTACAACACCGCCCGTCAACACCATGGGGAAGTTGATTGCTCCAGGAAAGTAAGCTAAGCTTAACCCTTCGGGGATGGCGGTACCACG |
| D8-2 | *Zobellela* sp. D8-2 | | TGCAGTCGAGCGGCAGCGGGAAAGTAGCTTGCTACTTTTGCCGGCGAGCGGCGGACGGGTGAGTAAGGCTTGGGTATCTGCCCAGTCGAGGGGGATAACAGTTGGAAACGACTGCTAATACCGCATACGCCCTACGGGGGAAAGGAGGGGATCTTCGGACCTTTCGCGATTGGATGAGCCCAAGTGAGATTAGCTAGTTGGTGAGGTAACGGCTCACCAAGGCGACGATCTCTAGCTGGTCTGAGAGGATGACCAGCCACACTGGGACTGAGACACGGCCCAGACTCCTACGGGAGGCAGCAGTGGGGAATATTGCACAATGGGGGAAACCCTGATGCAGCCATGCCGCGTGTGTGAAGAAGGCCTTCGGGTTGTAAAGCACTTTCAGTGGTGAGGAAGGAAGTAAGGTTAATACCCTTGCTTATTGACGTTAGCCACAGAAGAAGCACCGGCTAACTCCGTGCCAGCAGCCGCGGTAATACGGAGGGTGCAAGCGTTAATCGGAATTACTGGGCGTAAAGCGCACGCAGGCGGTTTGTTAAGCCAGATGTGAAAGCCCCGGGCTCAACCTGGGAATTGCATTTGGAACTGACAAACTAGAGTCTTGGAGAGGGGGGTAGAATTTCCGGTGTAGCGGTGAAATGCGTAGAGATCGGAAGGAATACCAGTGGCGAAGGCGGCCCCCTGGCCAAAGACTGACGCTCAGGTGCGAAAGCGTGGGGAGCAAACAGGATTAGATACCCTGGTAGTCCACGCTGTAAACGATGTCAACTTGGAGTCTGTGCCTATTGAGCGCGGGTTCCGGAGCTAACGCGTTAAGTTGACCGCCTGGGGAGTACGGCCGCAAGGTTAAAACTCAAATGAATTGACGGGGGCCCGCACAAGCGGTGGAGCATGTGGTTTAATTCGATGCAACGCGAAGAACCTTACCTACCCTTGACATAGTAAGAACTTGGCAGAGATGCCTTGGTGCCTTCGGGAACTTACATACAGGTGCTGCATGGCTGTCGTCAGCTCGTGTCGTGAGATGTTGGGTTAAGTCCCGCAACGAGCGCAACCCTTGTCCTTTGTTGCCAGCGATTCGGTCGGGAACTCAAAGGAGACTGCCGGTGATAAACCGGAGGAAGGTGGGGACGACGTCAAGTCATCATGGCCCTTACGGGTAGGGCTACACACGTGCTACAATGGCGCGTACAGAGGGCGGCGAACCTGCGAAGGTAAGCGAATCCCAAAAAGCGCGTCGTAGTCCGGATCGGAGTCTGCAACTCGACTCCGTGAAGTCGGAATCGCTAGTAATCGCAAATCAGAATGTTGCGGTGAATACGTTCCCGGGCCTTGTACACACCGCCCGTCACACCATGGGAGTGGGTTGCTCCAGAAGTAGATAGCTTAACCTTCGGGAGGGCGTTACCA |
| D8-3 | *Bacillus* sp. D8-3 | | GCTATACATGCAGTCGAGCGAACCGATTAAGAGCTTGCTCTTAAGAAGTTAGCGGCGGACGGGTGAGTAACACGTGGGTAACCTGCCCATAAGACTGGGATAACTCCGGGAAACCGGGGCTAATACCGGATAGCATTTTGAACCGCATGGTTCGAAATTGAAAGGCGGCTTCGGCTGTCACTTATGGATGGACCCGCGTCGCATTAGCTAGTTGGTGAGGTAACGGCTCACCAAGGCGACGATGCGTAGCCGACCTGAGAGGGTGATCGGCCACACTGGGACTGAGACACGGCCCAGACTCCTACGGGAGGCAGCAGTAGGGAATCTTCCGCAATGGACGAAAGTCTGACGGAGCAACGCCGCGTGAACGATGAAGGCCTTCGGGTCGTAAAGTTCTGTTGTTAGGGAAGAACAAGTATAAGTTGAATAAGCTTGTACCTTGACGGTACCTAACCAGAAAGCCACGGCTAACTACGTGCCAGCAGCCGCGGTAATACGTAGGTGGCAAGCGTTATCCGGAATTATTGGGCGTAAAGCGCGCGCAGGTGGTTTCTTAAGTCTGATGTGAAAGCCCACGGCTCAACCGTGGAGGGTCATTGGAAACTGGGAAACTTGAGTGCAGAAGAGGAAAGTGGAATTCCATGTGTAGCGGTGAAATGCGTAGAGATATGGAGGAACACCAGTGGCGAAGGCGACTTTCTGGTCTGTAACTGACACTGAGGCGCGAAAGCGTGGGGAGCAAACAGGATTAGATACCCTGGTAGTCCACGCCGTAAACGATGAGTGCTAAGTGTTAGGGGGTTTCCGCCCCTTAGTGCTGAAGTTAACGCATTAAGCACTCCGCCTGGGGAGTACGGCCGCAAGGCTGAAACTCAAAGGAATTGACGGGGGCCCGCACAAGCGGTGGAGCATGTGGTTTAATTCGAAGCAACGCGAAGAACCTTACCAGGTCTTGACATCCTCTGACAACCCTAGAGATAGGGCTTTCCCTTCGGGGACAGAGTGACAGGTGGTGCATGGTTGTCGTCAGCTCGTGTCGTGAGATGTTGGGTTAAGTCCCGCAACGAGCGCAACCCTTGATCTTAGTTGCCATCATTAAGTTGGGCACTCTAAGGTGACTGCCGGTGACAAACCGGAGGAAGGTGGGGATGACGTCAAATCATCATGCCCCTTATGACCTGGGCTACACACGTGCTACAATGGACGGTACAAAGAGCTGCAAGACCGCGAGGTGGAGCTAATCTCATAAAACCGTTCTCAGTTCGGATTGTAGGCTGCAACTCGCCTACATGAAGCTGGAATCGCTAGTAATCGCAGATCAGCATGCTGCGGTGAATACGTTCCCGGGCCTTGTACACACCGCCCGTCACACCACGAGAGTTTGTAACACCCGAAGTCGGTGGGGTAACCTTTGGAGCCAGCCGCCTAAG |
| D6-3 | *Ectopseudomonas* sp. D6-3 | | TGCAGTCGAGCGGATGAAGGGAGCTTGCTCCTGGATTCAGCGGCGGACGGGTGAGTAATGCCTAGGAATCTGCCTGGTAGTGGGGGATAACGTTCCGAAAGGAACGCTAATACCGCGTACGTCCTACGGGAGAAAGCAGGGGACCTTCGGGCCTTGCGCTATCAGATGAGCCTAGGTCGGATTAGCTAGTTGGTGAGGTAATGGCTCACCAAGGCGACGATCCGTAACTGGTCTGAGAGGATGATCAGTCACACTGGAACTGAGACACGGTCCAGACTCCTACGGGAGGCAGCAGTGGGGAATATTGGACAATGGGCGAAAGCCTGATCCAGCCATGCCGCGTGTGTGAAGAAGGTCTTCGGATTGTAAAGCACTTTAAGTTGGGAGGAAGGGCTGTAGGCTAATATCCTGCGGTTTTGACGTTACCAACAGAATAAGCACCGGCTAACTTCGTGCCAGCAGCCGCGGTAATACGAAGGGTGCAAGCGTTAATCGGAATTACTGGGCGTAAAGCGCGCGTAGGTGGTTCGTTAAGTTGGATGTGAAAGCCCCGGGCTCAACCTGGGAACTGCATCCAAAACTGGCGAGCTAGAGTACGGTAGAGGGTAGTGGAATTTCCTGTGTAGCGGTGAAATGCGTAGATATAGGAAGGAACACCAGTGGCGAAGGCGACTACCTGGACTGATACTGACACTGAGGTGCGAAAGCGTGGGGAGCAAACAGGATTAGATACCCTGGTAGTCCACGCCGTAAACGATGTCAACTAGCCGTTGGAATCCTTGAGATTTTAGTGGCGCAGCTAACGCATTAAGTTGACCGCCTGGGGAGTACGGCCGCAAGGTTAAAACTCAAATGAATTGACGGGGGCCCGCACAAGCGGTGGAGCATGTGGTTTAATTCGAAGCAACGCGAAGAACCTTACCTGGCCTTGACATGCTGAGAACTTTCCAGAGATGGATTGGTGCCTTCGGGAACTCAGACACAGGTGCTGCATGGCTGTCGTCAGCTCGTGTCGTGAGATGTTGGGTTAAGTCCCGTAACGAGCGCAACCCTTGTCCTTAGTTACCAGCACGTTATGGTGGGCACTCTAAGGAGACTGCCGGTGACAAACCGGAGGAAGGTGGGGATGACGTCAAGTCATCATGGCCCTTACGGCCAGGGCTACACACGTGCTACAATGGTCGGTACAAAGGGTTGCCAAGCCGCGAGGTGGAGCTAATCCCATAAAACCGATCGTAGTCCGGATCGCAGTCTGCAACTCGACTGCGTGAAGTCGGAATCGCTAGTAATCGTGAATCAGAATGTCACGGTGAATACGTTCCCGGGCCTTGTACACACCGCCCGTCACACCATGGGAGTGGGTTGCTCCAGAAGTAGCTAGTCTAACCTTCGGGGGGACGGTACCA |
| D9-1 | *Lysinibacillus* sp. D9-1 | | TGCAAGTCGAGCGAACAGAGAAGGAGCTTGCTCCTTTGACGTTAGCGGCGGACGGGTGAGTAACACGTGGGCAACCTACCTTATAGTTTGGGATAACTCCGGGAAACCGGGGCTAATACCGAATAATCTATTTTACTTCATGGTGAAATACTGAAAGACGGTTTCGGCTGTCGCTATAAGATGGGCCCGCGGCGCATTAGCTAGTTGGTGAGGTAACGGCTCACCAAGGCGACGATGCGTAGCCGACCTGAGAGGGTGATCGGCCACACTGGGACTGAGACACGGCCCAGACTCCTACGGGAGGCAGCAGTAGGGAATCTTCCACAATGGGCGAAAGCCTGATGGAGCAACGCCGCGTGAGTGAAGAAGGTTTTCGGATCGTAAAACTCTGTTGTAAGGGAAGAACAAGTACAGTAGTAACTGGCTGTACCTTGACGGTACCTTATTAGAAAGCCACGGCTAACTACGTGCCAGCAGCCGCGGTAATACGTAGGTGGCAAGCGTTGTCCGGAATTATTGGGCGTAAAGCGCGCGCAGGCGGTCCTTTAAGTCTGATGTGAAAGCCCACGGCTCAACCGTGGAGGGTCATTGGAAACTGGGGGACTTGAGTGCAGAAGAGGAAAGTGGAATTCCAAGTGTAGCGGTGAAATGCGTAGAGATTTGGAGGAACACCAGTGGCGAAGGCGACTTTCTGGTCTGTAACTGACGCTGAGGCGCGAAAGCGTGGGGAGCAAACAGGATTAGATACCCTGGTAGTCCACGCCGTAAACGATGAGTGCTAAGTGTTAGGGGGTTTCCGCCCCTTAGTGCTGCAGCTAACGCATTAAGCACTCCGCCTGGGGAGTACGGTCGCAAGACTGAAACTCAAAGGAATTGACGGGGGCCCGCACAAGCGGTGGAGCATGTGGTTTAATTCGAAGCAACGCGAAGAACCTTACCAGGTCTTGACATCCCGTTGACCCTGTAGAGATATGGTTTTCCCTTCGGGGACAACGGTGACAGGTGGTGCATGGTTGTCGTCAGCTCGTGTCGTGAGATGTTGGGTTAAGTCCCGCAACGAGCGCAACCCTTGATCTTAGTTGCCATCATTTAGTTGGGCACTCTAAGGTGACTGCCGGTGACAAACCGGAGGAAGGTGGGGATGACGTCAAATCATCATGCCCCTTATGACCTGGGCTACACACGTGCTACAATGGACGATACAAACGGTTGCCAACTCGCGAGAGGGAGCTAATCCGATAAAGTCGTTCTCAGTTCGGATTGTAGGCTGCAACTCGCCTACATGAAGCCGGAATCGCTAGTAATCGCGGATCAGCATGCCGCGGTGAATACGTTCCCGGGCCTTGTACACACCGCCCGTCACACCACGAGAGTTTGTAACACCCGAAGTCGGTGGGGTAACCTTTTGGAGCCAGCCGCCGAA |
| E1-3 | Stenotrophomonas  sp. E1-3 | | TGCAGTCGAACGGCAGCACAAAGGAGCTTGCTCCTTGGGTGGCGAGTGGCGGACGGGTGAGGAATACATCGGAATCTACTTTTTCGTGGGGGATAACGTAGGGAAACTTACGCTAATACCGCATACGACCTACGGGTGAAAGCAGGGGATCTTCGGACCTTGCGCGATTGAATGAGCCGATGTCGGATTAGCTAGTTGGCGGGGTAAAGGCCCACCAAGGCGACGATCCGTAGCTGGTCTGAGAGGATGATCAGCCACACTGGAACTGAGACACGGTCCAGACTCCTACGGGAGGCAGCAGTGGGGAATATTGGACAATGGGCGCAAGCCTGATCCAGCCATACCGCGTGGGTGAAGAAGGCCTTCGGGTTGTAAAGCCCTTTTGTTGGGAAAGAAATCCAGCCGGCTAATACCTGGTTGGGATGACGGTACCCAAAGAATAAGCACCGGCTAACTTCGTGCCAGCAGCCGCGGTAATACGAAGGGTGCAAGCGTTACTCGGAATTACTGGGCGTAAAGCGTGCGTAGGTGGTTGTTTAAGTCTGTTGTGAAAGCCCTGGGCTCAACCTGGGAACTGCAGTGGAAACTGGACGACTAGAGTGTGGTAGAGGGTAGCGGAATTCCTGGTGTAGCAGTGAAATGCGTAGAGATCAGGAGGAACATCCATGGCGAAGGCAGCTACCTGGACCAACACTGACACTGAGGCACGAAAGCGTGGGGAGCAAACAGGATTAGATACCCTGGTAGTCCACGCCCTAAACGATGCGAACTGGATGTTGGGTGCAATTTGGCACGCAGTATCGAAGCTAACGCGTTAAGTTCGCCGCCTGGGGAGTACGGTCGCAAGACTGAAACTCAAAGGAATTGACGGGGGCCCGCACAAGCGGTGGAGTATGTGGTTTAATTCGATGCAACGCGAAGAACCTTACCTGGCCTTGACATGTCGAGAACTTTCCAGAGATGGATTGGTGCCTTCGGGAACTCGAACACAGGTGCTGCATGGCTGTCGTCAGCTCGTGTCGTGAGATGTTGGGTTAAGTCCCGCAACGAGCGCAACCCTTGTCCTTAGTTGCCAGCACGTAATGGTGGGAACTCTAAGGAGACCGCCGGTGACAAACCGGAGGAAGGTGGGGATGACGTCAAGTCATCATGGCCCTTACGGCCAGGGCTACACACGTACTACAATGGTAGGGACAGAGGGCTGCAAGCCGGCGACGGTAAGCCAATCCCAGAAACCCTATCTCAGTCCGGATTGGAGTCTGCAACTCGACTCCATGAAGTCGGAATCGCTAGTAATCGCAGATCAGCATTGCTGCGGTGAATACGTTCCCGGGCCTTGTACACACCGCCCGTCACACCATGGGAGTTTGTTGCACCAGAAGCAGGTAGCTTAACCTTCGGGAGGGCGCTGCCAC |
| E1-YE2 | *Stenotrophomonas* sp. E1-YE2 | | TGCAGTCGAACGGCAGCACAGTAAGAGCTTGCTCTTATGGGTGGCGAGTGGCGGACGGGTGAGGAATACATCGGAATCTACCTTTTCGTGGGGGATAACGTAGGGAAACTTACGCTAATACCGCATACGACCTTCGGGTGAAAGCAGGGGACCTTCGGGCCTTGCGCGGATAGATGAGCCGATGTCGGATTAGCTAGTTGGCGGGGTAAAGGCCCACCAAGGCGACGATCCGTAGCTGGTCTGAGAGGATGATCAGCCACACTGGAACTGAGACACGGTCCAGACTCCTACGGGAGGCAGCAGTGGGGAATATTGGACAATGGGCGCAAGCCTGATCCAGCCATACCGCGTGGGTGAAGAAGGCCTTCGGGTTGTAAAGCCCTTTTGTTGGGAAAGAAAAGCAGCTGGCTAATACCCGGTTGTTCTGACGGTACCCAAAGAATAAGCACCGGCTAACTTCGTGCCAGCAGCCGCGGTAATACGAAGGGTGCAAGCGTTACTCGGAATTACTGGGCGTAAAGCGTGCGTAGGTGGTTGTTTAAGTCTGTTGTGAAAGCCCTGGGCTCAACCTGGGAATTGCAGTGGATACTGGGCGACTAGAGTGTGGTAGAGGGTAGTGGAATTCCCGGTGTAGCAGTGAAATGCGTAGAGATCGGGAGGAACATCCATGGCGAAGGCAGCTACCTGGACCAACACTGACACTGAGGCACGAAAGCGTGGGGAGCAAACAGGATTAGATACCCTGGTAGTCCACGCCCTAAACGATGCGAACTGGATGTTGGGTGCAATTTGGCACGCAGTATCGAAGCTAACGCGTTAAGTTCGCCGCCTGGGGAGTACGGTCGCAAGACTGAAACTCAAAGGAATTGACGGGGGCCCGCACAAGCGGTGGAGTATGTGGTTTAATTCGATGCAACGCGAAGAACCTTACCTGGTCTTGACATGTCGAGAACTTTCCAGAGATGGATTGGTGCCTTCGGGAACTCGAACACAGGTGCTGCATGGCTGTCGTCAGCTCGTGTCGTGAGATGTTGGGTTAAGTCCCGCAACGAGCGCAACCCTTGTCCTTAGTTGCCAGCACGTAATGGTGGGAACTCTAAGGAGACCGCCGGTGACAAACCGGAGGAAGGTGGGGATGACGTCAAGTCATCATGGCCCTTACGACCAGGGCTACACACGTACTACAATGGTAGGGACAGAGGGCTGCAAACCCGCGAGGGCAAGCCAATCCCAGAAACCCTATCTCAGTCCGGATTGGAGTCTGCAACTCGACTCCATGAAGTCGGAATCGCTAGTAATCGCAGATCAGCATTGCTGCGGTGAATACGTTCCCGGGCCTTGTACACACCGCCCGTCACACCATGGGAGTTTGTTGCACCAGAAGCAGGTAGCTTAACCTTCGGGAGGGCGCTG |
| E1-LB2 | *Aeromonas* sp. E1-LB2 | | TGCAGTCGAGCGGCAGCGGGAAAGTAGCTTGCTACTTTTGCCGGCGAGCGGCGGACGGGTGAGTAATGCCTGGGGATCTGCCCAGTCGAGGGGGATAACAGTTGGAAACGACTGCTAATACCGCATACGCCCTACGGGGGAAAGGAGGGGACCTTCGGGCCTTTCGCGATTGGATGAACCCAGGTGGGATTAGCTAGTTGGTGGGGTAATGGCTCACCAAGGCGACGATCCCTAGCTGGTCTGAGAGGATGATCAGCCACACTGGAACTGAGACACGGTCCAGACTCCTACGGGAGGCAGCAGTGGGGAATATTGCACAATGGGGGAAACCCTGATGCAGCCATGCCGCGTGTGTGAAGAAGGCCTTCGGGTTGTAAAGCACTTTCAGCGAGGAGGAAAGGTTGGCGCCTAATACGTGTCAACTGTGACGTTACTCGCAGAAGAAGCACCGGCTAACTCCGTGCCAGCAGCCGCGGTAATACGGAGGGTGCAAGCGTTAATCGGAATTACTGGGCGTAAAGCGCACGCAGGCGGTTGGATAAGTTAGATGTGAAAGCCCCGGGCTCAACCTGGGAATTGCATTTAAAACTGTCCAGCTAGAGTCTTGTAGAGGGGGGTAGAATTCCAGGTGTAGCGGTGAAATGCGTAGAGATCTGGAGGAATACCGGTGGCGAAGGCGGCCCCCTGGACAAAGACTGACGCTCAGGTGCGAAAGCGTGGGGAGCAAACAGGATTAGATACCCTGGTAGTCCACGCCGTAAACGATGTCGATTTGGAGGCTGTGTCCTTGAGACGTGGCTTCCGGAGCTAACGCGTTAAATCGACCGCCTGGGGAGTACGGCCGCAAGGTTAAAACTCAAATGAATTGACGGGGGCCCGCACAAGCGGTGGAGCATGTGGTTTAATTCGATGCAACGCGAAGAACCTTACCTGGCCTTGACATGTCTGGAATCCTGTAGAGATACGGGAGTGCCTTCGGGAATCAGAACACAGGTGCTGCATGGCTGTCGTCAGCTCGTGTCGTGAGATGTTGGGTTAAGTCCCGCAACGAGCGCAACCCCTGTCCTTTGTTGCCAGCACGTAATGGTGGGAACTCAAGGGAGACTGCCGGTGATAAACCGGAGGAAGGTGGGGATGACGTCAAGTCATCATGGCCCTTACGGCCAGGGCTACACACGTGCTACAATGGCGCGTACAGAGGGCTGCAAGCTAGCGATAGTGAGCGAATCCCAAAAAGCGCGTCGTAGTCCGGATCGGAGTCTGCAACTCGACTCCGTGAAGTCGGAATCGCTAGTAATCGCGAATCAGAATGTCGCGGTGAATACGTTCCCGGGCCTTGTACACACCGCCCGTCACACCATGGGAGTGGGTTGCACCAGGAAAGTAGATAGCTTAACCTTCGGGAGGGCGTT |

**Table S2.** One-way ANOVA table for analyzing the plant growth-promoting effects of alginate-degrading bacteria on *Arabidopsis thaliana*, as shown in Figure 7. Statistical significance was evaluated using Tukey’s Honest Significant Difference (HSD) test.

| **Parameter** | **Source** | **DF** | **Sum of Square** | **Mean Square** | **F Statistic** | **P-value** |
| --- | --- | --- | --- | --- | --- | --- |
| Fresh weight | Groups | 6 | 0.1529 | 0.02549 | 12.3298 | 9.19e-7 |
|  | Error | 28 | 0.05789 | 0.002067 |  |  |
|  | Total | 34 | 0.2108 | 0.006201 |  |  |
| Dry weight | Groups | 6 | 0.0003646349 | 0.00006077248 | 4.1031591 | 0.004456789 |
|  | Error | 28 | 0.000414712 | 0.00001481114 |  |  |
|  | Total | 34 | 0.0007793469 | 0.00002292197 |  |  |
| Number of leaves | Groups | 6 | 14.5714286 | 2.4285714 | 1.0240966 | 0.4303301 |
|  | Error | 28 | 66.3999855 | 2.3714281 |  |  |
|  | Total | 34 | 80.9714141 | 2.3815122 |  |  |
| Total leaf area | Groups | 6 | 304.7493 | 50.7915 | 10.936 | 0.000002861 |
|  | Error | 28 | 130.0441 | 4.6444 |  |  |
|  | Total | 34 | 434.7933 | 12.788 |  |  |
